# Automated breast tumor ultrasound image segmentation with hybrid UNet and classification using fine-tuned CNN model

**DOI:** 10.1016/j.heliyon.2023.e21369

**Published:** 2023-10-21

**Authors:** Shahed Hossain, Sami Azam, Sidratul Montaha, Asif Karim, Sadia Sultana Chowa, Chaity Mondol, Md Zahid Hasan, Mirjam Jonkman

**Affiliations:** a Health Informatics Research Laboratory (HIRL), Department of Computer Science and Engineering, Daffodil International University, Dhaka, 1341, Bangladesh; b Faculty of Science and Technology, Charles Darwin University, Casuarina, 0909, NT, Australia; c Department of Computer Science, University of Calgary, Calgary, AB, T2N 1N4, Canada

**Keywords:** Image preprocessing, Spatial self attention, Channel self attention, Hybrid attention module, Image segmentation, Deep learning

## Abstract

**Introduction:**

Breast cancer stands as the second most deadly form of cancer among women worldwide. Early diagnosis and treatment can significantly mitigate mortality rates.

**Purpose:**

The study aims to classify breast ultrasound images into benign and malignant tumors. This approach involves segmenting the breast's region of interest (ROI) employing an optimized UNet architecture and classifying the ROIs through an optimized shallow CNN model utilizing an ablation study.

**Method:**

Several image processing techniques are utilized to improve image quality by removing text, artifacts, and speckle noise, and statistical analysis is done to check the enhanced image quality is satisfactory. With the processed dataset, the segmentation of breast tumor ROI is carried out, optimizing the UNet model through an ablation study where the architectural configuration and hyperparameters are altered. After obtaining the tumor ROIs from the fine-tuned UNet model (RKO-UNet), an optimized CNN model is employed to classify the tumor into benign and malignant classes. To enhance the CNN model's performance, an ablation study is conducted, coupled with the integration of an attention unit. The model's performance is further assessed by classifying breast cancer with mammogram images.

**Result:**

The proposed classification model (RKONet-13) results in an accuracy of 98.41 %. The performance of the proposed model is further compared with five transfer learning models for both pre-segmented and post-segmented datasets. K-fold cross-validation is done to assess the proposed RKONet-13 model's performance stability. Furthermore, the performance of the proposed model is compared with previous literature, where the proposed model outperforms existing methods, demonstrating its effectiveness in breast cancer diagnosis. Lastly, the model demonstrates its robustness for breast cancer classification, delivering an exceptional performance of 96.21 % on a mammogram dataset.

**Conclusion:**

The efficacy of this study relies on image pre-processing, segmentation with hybrid attention UNet, and classification with fine-tuned robust CNN model. This comprehensive approach aims to determine an effective technique for detecting breast cancer within ultrasound images.

## 1 Introduction

**Fig. 1:**
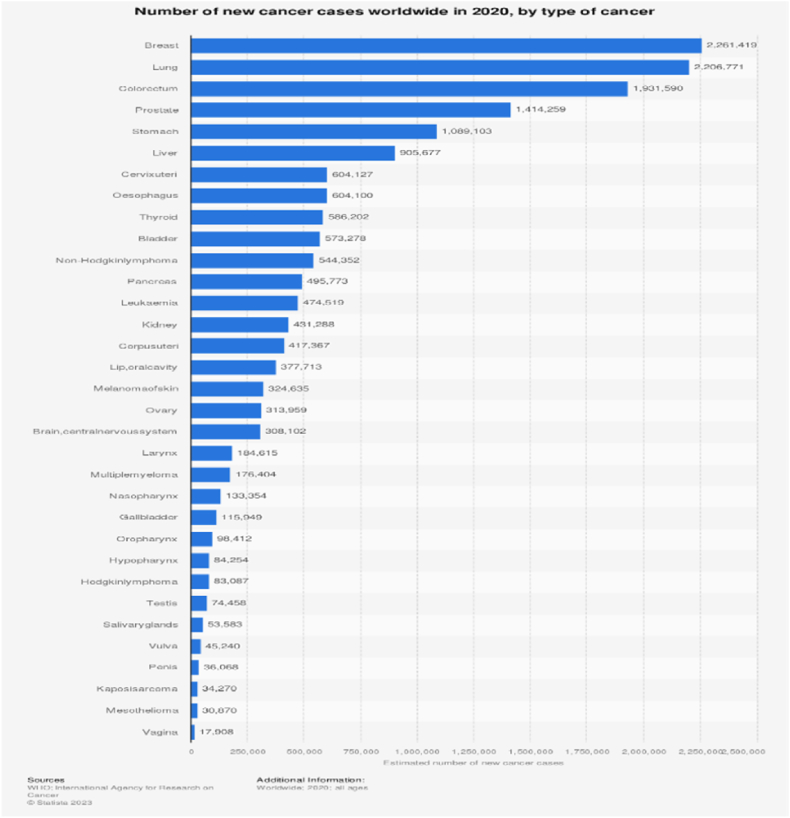
The expected total number of new cancer cases by cancer type for the year 2020 is shown in this statistic. It is estimated that there are around 18.1 million new instances of cancer worldwide, affecting people of all sexes and ages. The most common kind of cancer globally, affecting 2.2 million new cases, was breast cancer.

Breast cancer is one of the prominent causes of female fatalities on a global scale [1], with approximately 500000 annual deaths [2]. It is the most widespread cancer in women worldwide [3]. Research has shown that older women are more prone to weakness, as seen by a greater risk of prefrailty and frailty. Furthermore, breast cancer mortality rates are increased for women over the age of 55 [4,5]. In the adherence to early identification of breast cancer, assessing disease burden may aid the experts in monitoring progression and revealing the health imbalances [6]. The highest breast cancer rates are seen in developed countries: 74.1 new circumstances per 100,000 females, compared to 31.3 new cases per 100,000 in less developed countries [7]. The increase of new breast cancer cases was reported at 1.67 million in 2012 [8], 1.8 million in 2013 [9], and about 2.2 million in 2020, shown in Fig. 1, 12.5 % of the total cancer cases. It has overtaken lung cancer and become the most diagnosed cancer worldwide [10,11]. By 2025, the World Health Organization (WHO) estimates that 19.3 million additional cases of cancer will be diagnosed globally [12] and 27.5 million by 2040 with 16.3 million deaths [13]. Though health information is easy to access through social media, a lack of study on improved crisis communication in these platforms may make it more difficult to effectively notify the public about critical information [14,15], which may impede early breast cancer diagnosis. To increase public understanding of early detection techniques and their importance, clear communication is essential. Moreover, integrating social media and internet usage has not only strengthened communication but also, with innovations, brought transformative potential to various fields like medical research.

According to prior studies [16,17], developing mental issues such as anxiety and stress [18] is higher in breast cancer patients. Early-stage breast cancer detection can reduce mortality [19] and significantly increase the survival chances [20]. However, it is difficult to identify breast cancer in its early stages. Breast cancer screening and diagnosis are time-consuming, and the presence of noise, artifacts, and other difficulties creates difficulties for a radiologist to classify the medical images. In addition, there is a global shortage of radiologists and medical professionals who can interpret screening data, particularly in rural regions and developing nations [21]. The increase in the number of patients adds to the burden of radiologists, which may contribute to misdiagnosis. In addition to mammograms, radiologists typically use ultrasound images to diagnose patients. This takes time and might lead to poor outcomes if the radiologist is not properly trained. On the other hand, during the COVID-19 epidemic, breast cancer patients experienced delays in tests, treatments, and operations [[22], [23], [24], [25]]. In addition to causing delays in breast cancer tests, therapies, and operations, the COVID-19 pandemic's disruption of the medical supply chains showed flaws in depending entirely on conventional approaches [[26], [27], [28]]. This crisis highlighted the significance of implementing digital platforms for telemedicine and remote patient monitoring, diversifying supply sources, and encouraging collaborations between healthcare institutions and technology providers to build a resilient and adaptable supply chain ecosystem for ongoing and uninterrupted breast cancer care [29].

Currently, early detection of breast cancer is distinguished through diverse medical imaging modalities such as ultrasound, magnetic resonance imaging (MRI), and mammography [[30], [31], [32]]. Ultrasound has better sensitivity for dense breasts and can be better for differentiating solid tumors from cysts than mammography [33]. Ultrasound technology is less expensive than mammography and may detect changes that are not visible in mammograms [[34], [35], [36]]. Radiologists can distinguish between various tissues. Segmenting breast ultrasound images may be valuable for tumor localization and breast cancer diagnosis [37]. However, scrutiny of breast ultrasound images depends on the radiologist's clinical expertise and involves subjectivity and inter-observer variability. It is also time-consuming. A computer-aided detection (CAD) system may eliminate inconsistency and reduce the workload of radiologists by assisting in the analysis of breast ultrasound images [[38], [39], [40]]. It might also be used to segment breast images automatically [41]. Previous CAD systems generally relied on manually created visual information that posed difficulties in generalizing ultrasound images acquired from diverse techniques [[42], [43], [44], [45], [46], [47]]. The development of artificial intelligence (AI) technology for the automated detection of breast cancers using ultrasound images has been aided by some recent breakthroughs [[48], [49], [50]]. Deep learning can have benefits for medical image analysis, including breast cancer [51]. CNN is a kind of deep learning that has several layer hierarchies and translates the pixels of an image into features. These features are used for image segmentation, disease identification, and classification. Deep Learning technology can help to identify breast cancer early and reduce ultrasound interpretation time [[52], [53], [54]]. On the other hand, by enhancing early detection, lowering the demand on screening resources, and optimizing surgical planning, a deep learning breast cancer detector CAD system can mitigate supply chain issues and increase overall efficiency and resource allocation in breast cancer care during Covid-19 pandemic-related disruptions [[55], [56], [57]].

This study aims to segment, detect, and classify ultrasound images into benign and malignant tumors. All the experiments that are carried out in this study are done using the Breast Ultrasound Image (BUI) dataset. However, its images contain texts, speckle noise, and artifacts. Removal of text, speckle noise, and artifacts from the ultrasound images is imperative to obtain good performance from a CNN model. Various image preprocessing techniques are explored to eliminate artifacts and noises. Statistical analysis for the preprocessed images is performed to assess various image processing techniques, including Mean Squared Error (MSE), Peak signal-to-noise ratio (PSNR), Root Mean Squared Error (RMSE), and Structural Similarity Index Measure (SSIM). This study proposes two completely automated and reliable deep learning models, RKO-UNet and RkoNet-13, based on an UNet-like architecture and a Base CNN model, respectively. Expanded UNet is employed for image segmentation and RKONet-13 for segmented image classification. Both models are fine-tuned through ablation studies. The overall process of ultrasound image segmentation and classification proposed in this study is shown in Fig. 5. Five pre-trained and fine-tuned transfer learning models are employed to compare the performance and robustness of the proposed classification model with other techniques. Several performance metrics are used to evaluate both models. The segmentation model's effectiveness, robustness, and accuracy are evaluated using the PSNR, SSIM, Dice Similarity, Normalized Cross Correlation, Normalized Mutual Information, Sum of Squared Difference, RMSE, MSE, Feature-Based Similarity Index, and Universal Image Quality Index. The classification model is evaluated using several metrics, including precision, recall, F1 score, specificity, and sensitivity. K-fold cross-validation is conducted for the proposed model to ensure overfitting is not occurring. Subsequently, this study uses an additional breast cancer mammogram dataset to substantiate the resilience and effectiveness of the proposed CNN model. It demonstrates the capability of the proposed CNN model (RKONet-13) to identify breast tumors and detect breast cancer at the same time. Furthermore, it proves its proficiency in detecting breast cancer from ultrasound images and mammogram images. The strategy of the work can be described as follows.

1. Initially, noise diminution and data pre-processing algorithms are used to remove text, artifacts, and spackle noise from the raw ultrasound images.
2. On the pre-processed images, statistical analysis is carried out using MSE, PSNR, SSIM, and RMSE to make sure that the information is not deteriorated and the quality of the image is still good.
3. A new model, referred as the Hybrid Attention UNet (RKO-UNet), is proposed. It is based on fine-tuning the UNet architecture and incorporating both spatial self-attention and channel self-attention.
4. An ablation study, modifying the segmentation model, is carried out to enhance the suggested model's performance.
5. A CNN model is built from scratch and modified by fine-tuning the parameters to find the best classifier.
6. RKONet-13 is recommended on a fine-tuned CNN architecture following experimentation with the segmented dataset, as it produces the most accurate classification.
7. Another ablation study is done to enhance the classification performance.

The main aim and contributions of the work can be summarized as follows.

1. To develop an automated CAD system that can segment and classify breast tumor.
2. To develop a hybrid attention UNet (RKO-UNet) model that can segment the ROI from the breast ultrasound images.
3. To develop a robust CNN (RKONet-13) can detect or classify breast cancer from the ROI images obtained from the UNet model.

The remaining section of this study's materials are structured as: the comprehensive and critical analyses of related work are presented in section 2, the materials and methods is described in section 3, the experiment results are shown in section 4, and the discussion and conclusion are given in part 5 and 6.

## 2 Related work

Over the years, several automated computer vision-based techniques have been developed to classify breast cancer based on ultrasound images [58,59], where some of them focus on segmentation [60].

Irfan et al. [31] established the Dilated Semantic Segmentation Network (Di-CNN) to identify and categorize breast cancer using the BUI Dataset and achieved 98.9 % accuracy. They applied a pre-trained DenseNet201 deep model, developed via transfer learning and utilized for feature extraction. With the trained model, 24-layered CNN were applied with fused feature data in parallel. The outcomes demonstrated that the fusion procedure increases recognition accuracy. Jianrui et al. [44] used a self-organizing map to map the instance space to the concept space, then distributed the instances of each bag in the concept space to construct the bag feature vector, and lastly, used a support vector machine to identify tumors with an accuracy of 91.07 %, (p 0.005) with an area under the receiver operator characteristic curve of 0.96 for the 168 images of the dataset. They used data collected at the Department of Ultrasound, Second Affiliated Hospital of Harbin Medical University. Zhemin et al. [61] utilize transfer learning models to extract features, select the best model, and then perform feature fusion and classification based on those features. They merged four datasets (BUSI, OMI, Dataset B, and Hospital) and obtained 95.48 % accuracy after experimenting with 1328 images. Tariq et al. [62] used an ensemble decision tree with the RUSBoost model to classify the OASBUD (Open Access Series of Breast Ultrasound Data) and BHE datasets separately, obtaining 99.98 % accuracy for the OASBUD dataset and 97.86 % accuracy for the BHE dataset. For OASBUD, the proposed system achieved confidence intervals (CI) of 91.48 %–99.38 %, and for the BHE dataset, CIs of 94.90 %–97.86 %. Mishra et al. [63] isolated the ROI in images from the BUSI dataset and extracted features using a machine learning radionics-based classification pipeline, resulting in a 97.4 % accuracy. Their method produced values for the F1-score, Mathew's correlation coefficient, and area under the curve of 97 %, 94 %, and 0.959, respectively. Kiran et al. [64] modified a pre-trained transfer learning model and extracted features using global average polling, then they fused the best features using RDE and RGW employing a probability-based serial approach and classified them using a machine learning model that achieved 99.1 % accuracy. Using a Transfer Learning Model with Deep Representation Scaling Layers, Byra et al. [65] developed a deep learning model that achieved 91.5 % accuracy and an AUC of 0.95 on the BUI dataset. Moon et al. [66] developed a CAD system using an ensemble CNN model (VGGNet, ResNet, and DenseNet) with two datasets, obtaining 91.10 % accuracy for a private dataset and 94.62 % for the BUSI dataset. They recorded 92.31 % sensitivity, 95.60 % specificity, 90 % precision, 91.14 % F1 score, respectively. Yang et al. [67] applied a deep learning-based network to 14043 images and achieved an accuracy on BUS images of 86.40 %, with an AUC of 0.913, a sensitivity of 88.84 %, and a specificity of 83.77 %. They used medical data from 32 hospitals. Xiangmin et al. [68] introduced a deep, doubly supervised transfer learning network for categorizing breast cancer using the bimodal breast Ultrasound dataset. It aimed to introduce the Maximum Mean Discrepancy (MMD) criterion-based Learning using Privileged Information (LUPI) paradigm. They integrated the two methods using a new doubly supervised TL network (DDSTN) and achieved 86.79 % accuracy. In Table 1, shows the categorization of the previous study with limitations.

**Table 1:** Shows the tabular view of the previous study.

Paper	Dataset	Classifier	Accuracy	Limitation
Irfan et al. [31]	BUI	Bi–CNN, transfer learning	98.9 %	Small dataset size, noisy predictions for erosion operation, lack of comprehensive geometric features extraction, computationally intensive, lack of enough validation of the results and experiments.
Jianrui et al. [44]	Department of Ultrasound, Second Affiliated Hospital of Harbin Medical University	Self-organizing map, Machine Learning Classifier	91.07 %	Focus on local texture features only, limited representation of global features, predominance of common tumor types in dataset.
Zhemin et al. [61]	1) BUSI 2) OMI 3) Dataset B 4) Hospital	adaptive spatial feature fusion	95.48 %	Reliance on transfer learning of deep features, lack of computational analysis.
Mishra et al. [63]	BUSI	ML Radiomics based Classifier	97.4 %	Limited dataset source (single centre), exclusively traditional image features.
Byra et al. [65]	BUSI	Hybrid Classifier (Transfer learning + scaling layers)	91.5 %	Limited fine-tuning of only last residual block, high computational requirements and potential overfitting.
Kiran et al. [64]	BUSI	Transfer learning with feature fusion, RDE, RGW, Machine learning Classifier	99.1 %	Limited number of datasets, high similarity of lesions
Moon et al. [66]	1) Private Dataset BUSI	Ensemble CNN (VGGNet, ResNet, DenseNet)	91.10 % 94.62 %	Small size of dataset, limited exploration of hyperparameters.
tariq et al. [62]	1) OASBUD 2) BHE	Ensemble Classifier, RUSBoost	99.98 % 97.86 %	Sensitivity of the dataset variation.
Yang et al. [67]	Collect from 32 Hospital	Deep Learning	86.40 %	Limited Generalizability
Xiangmin et al. [68]	Biomodal breast Ultrasound	DDSTN, MMD, LUPI	86.79 %	Imbalanced modalities distribution, limited evaluation metrics
Proposed Model	BUI	Robust CNN model (RKONet-13)	98.41	Limited number of images in dataset, less experiments with the model

## 3 Materials and methods

The section comprehensively explains the materials and methods employed in this study, providing insights into the dataset and its description, the overall procedure carried out in sequential order, image preprocessing and verification methods, and finally, the image segmentation and classification approaches.

### 3.1 Dataset description

The Breast Ultrasound Image (BUI) dataset from the open-source platform Kaggle is used for this research. A total of 780 ultrasound images are analyzed in this study. The images are categorized into three classes: 133 normal images, 210 malignant tumor images and 487 benign tumor images. The image dimension of this dataset is 500 × 500 pixels on average, and the images are in PNG format [69]. Table 2 provides an overview of the dataset description.

**Table 2:** Dataset illustration.

Name	Description
Image Numbers	780
Average Dimension	500 x 500
Color Grading	Grayscale
Image Format	PNG
Normal	133
Benign	487
Malignant	210

**Fig. 2:**
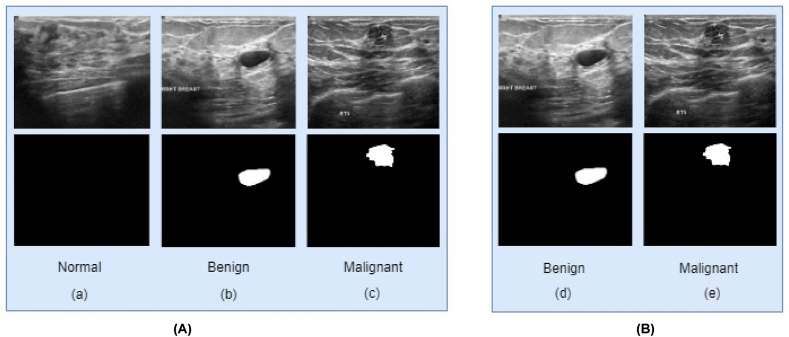
(A) images from the original dataset, and (B) the normal class has been removed manually. In this dataset, every image has a ground truth, and every ground truth has the same dimension, color grading, and data format as the original image. A sample of a mask image is also shown.

We manually removed the normal class, containing 133 images, from the dataset because this study essentially focused only on the breast tumors, cancerous cells, and its surrounding tissue. After removing the normal images, the dataset consists of two classes and the quantity of the images remain unchanged for these classes. Fig. 2 shows some examples of the two classes along with the characteristics and artifacts. 2(A) depicted the original dataset images with three classes. 2(B) show the dataset with two classes that is used in this study.

**Fig. 3:**
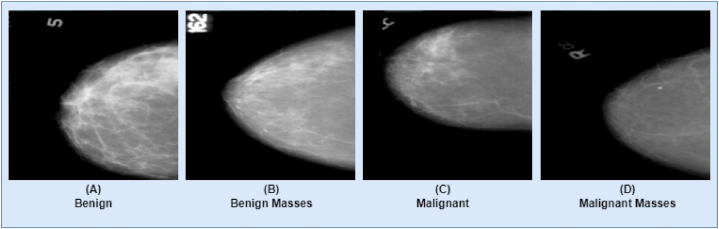
The CBIS-DDSM dataset comprises mammograms categorized into four classes, each exhibiting a range of artifacts across all classes. (A) Bening, (B) Bening Masses, (C) Malignant, (D) Malignant Masses.

This study uses an extra-best cancer mammogram dataset to check the robustness of the proposed model. The collection contains 1459 images with an average size of 224 x 224 [46]. The data is preserved in PNG format, while the color grading for the images is in RGB format. There are 417 images of benign masses and 398 images of malignant masses in the entire dataset. Additionally, there are 300 images categorized as benign and 344 images classified as malignant. Fig. 3 gives a visualization of the breast mammogram images from four different classes. Table 3 provides a brief explanation of the dataset.

**Table 3:** Dataset illustration.

Name	Description
Image Numbers	1459
Average Dimension	224 x 224
Color Grading	RGB
Image Format	PNG
Benign mass	417
Malignant mass	398
Benign	300
Malignant	344

#### 3.1.1 Merged ground truths

**Fig. 4:**
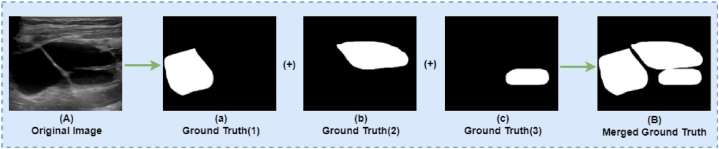
Merged Ground Truth: (A) shows the original images with multiple tumors. Ground truth (a), (b), and (c) are the ground truths of the original image (A), combining (a), (b), (c) gives the merged ground truth image (B).

In this context, ground truth refers to the precise boundary of breast tumors (both benign and malignant) in the ultrasound images, established using a freehand segmentation in MATLAB [69], which is used to train and validate segmentation models or algorithms. Some images have multiple ground truths (different annotations or labels of structures or regions of interest). Ground truths must be merged before segmentation because the segmentation model is trained by implementing a particular image's ground truths on that original image. This study merged all the ground truth for individual images. The output of merging multiple ground truths is shown in Fig. 4.

**Fig. 5:**
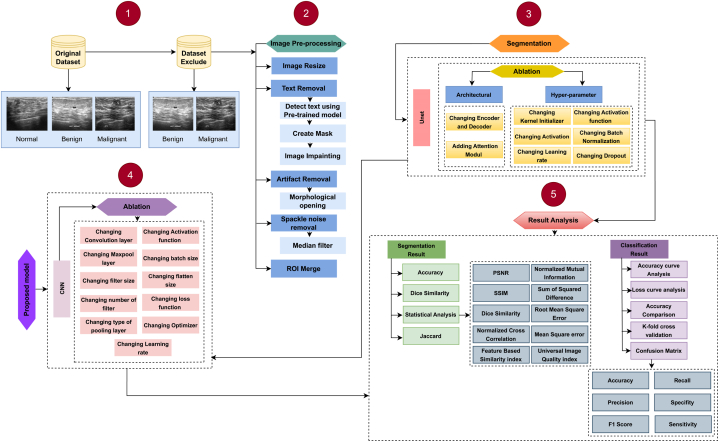
The workflow of this study is as follows: Frame (1) depicts some of the original ultrasound images of BUIS dataset. (2) Using different image preprocessing steps, a preprocessed dataset is generated. (3) The preprocessed dataset is segmented using segmentation techniques, resulting in a segmented dataset, produced by the UNet model. (4) Based on an ablation study, a more robust and accurate model is developed, and (5) the final model's results are analyzed using performance metrics.

### 3.2 Overview of methodology

This work aims to develop an efficient automated deep learning method to help radiologists accurately and quickly segment and classify benign and malignant tumors. The procedure is shown in Fig. 5.

In Fig. 5, it is shown that the initial phase involves employing noise mitigation and data pre-processing algorithms to eliminate text, artifacts, and speckle noise from the raw ultrasound images. Subsequently, on the pre-processed images, statistical inspection such as MSE, PSNR, SSIM, and RMSE, is conducted to make sure the information is not deteriorated and image quality is still good. Furthermore, a Hybrid Attention UNet (RKO-UNet) model is presented for the tumor segmentation, fine-tuning the UNet architecture and integrating both spatial self-attention and channel self-attention. An ablation study, modifying the segmentation model, is carried out to enhance the suggested model's performance. For the classification task, CNN model is built from scratch and modified by fine-tuning the parameters to find a superior classifier for the segmented ROIs. RKONet-13 is recommended on a fine-tuned CNN network following experimentation with the segmented dataset, as it produces the most accurate classification. Another ablation study is done to enhance the classification performance. The result of the final model is then analyzed using a set of performance metrics. The model's robustness is assessed by comparing its performance with five transfer learning models, establishing its competitive edge. Five-fold cross-validation is [70] done to determine whether the model is overfitted or not. The performance comparison with prior studies is also done. The proposed CNN model's performance for classification task is evaluated with a breast cancer mammogram image dataset and the model shows its supremacy in classifying breast cancer with dataset of other imaging modalities as well.

In summary, this methodology demonstrates the importance of segmentation and classification for breast cancer diagnosis, effectively addressing medical challenges. Also, a precise understanding on health-related stresses and anxieties [71] that can arise during such crises, especially for pandemic like COVID-19 is also discussed.

### 3.3 Image pre-processing techniques

**Fig. 6:**
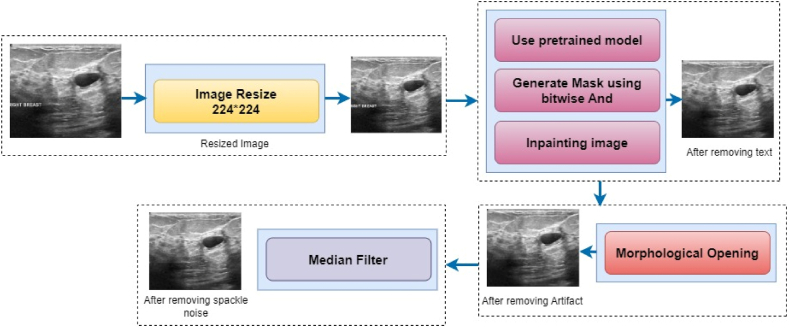
Image pre-processing steps.

Image pre-processing is probably the most prominent process before images are fed into a deep neural system. It helps to obtain good performance and reduce the computational time of a network [72,73]. Pre-processing aims to improve the image's quality, eliminating unwanted distortions, and improving important features [74]. Ultrasound images are affected by several types of noise. Ultrasound images contain low-resolution created by the ultrasound wave's reflection [75]. Without using pre-processing methods, it is tenacious for a neural network model to classify ultrasound images correctly. This section describes how the quality of ultrasound images can be improved with image resizing, text removal, artifact removal, and speckle noise removal. Fig. 6 illustrates the main steps of the image pre-processing done in this study, including image resize, text removal, artifacts removal, speckle noise removal.

The images are resized, and text is removed from the ultrasound images, using a pretrained model, followed by inpainting. Then artifacts are removed using morphological opening. After removing speckle noise, the pre-processed images are subjected to assessment methods such as MSE, PSNR, SSIM, and RMSE to assess the outcomes. The outcome image after each procedure is also shown in Fig. 6.

#### 3.3.1 Image resizing

Resizing images is a common procedure in image preprocessing in deep learning [72]. The average image resolution in BUI dataset is 512 X 512, but different images have different resolutions. All images are resized to 224 X 224.

#### 3.3.2 Text removal

Some images in the dataset contain unwanted text that can affect the model's performance. In this pre-processing step, an inpainting algorithm from OpenCV is employed, combined with a pre-trained OCR (Optical Character Recognition) model to remove text automatically. There are three steps: first, recognize text in the image and gain the bounding boxes, second, generate a mask and third paint in the areas with test. To acquire the bounding box of the text, Keras-OCR is used. Keras-OCR convey out-of-the-box OCR models and an end-to-end training pipeline to build new OCR models [76]. It automatically downloads the pre-trained weights for the detector and the recognizer. The pre-trained model is used in this study as it works well. While passing an image through Keras-OCR, a tuple (word, box) will be returned, where the box comprises the coordinates (x, y) of the word's four corner boxes. After recognizing the text, a mask of the same size as the input images which contains only the text is generated. This informs the algorithm which area of the image needs to be painted in. Finally, an inpainting algorithm is used to paint in the image's masked regions. We used cv2.INPAINT_NS [77]. The algorithm makes used of partial differential equations and is based on fluid dynamics. After inpainting, the output image is presented without any text. Image 6 shows the whole process of removing text from an image.

#### 3.3.3 Artifact removal

**Fig. 7:**
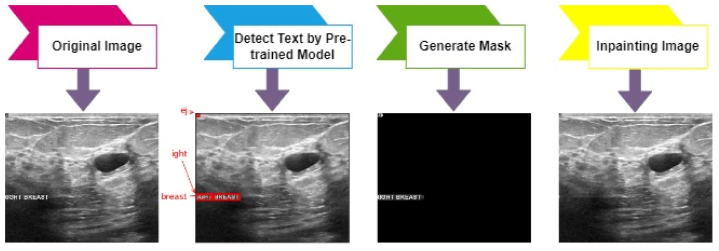
Text removal process, step by step.

Inadvertently, undesirable items or regions may sometimes appear in the images. As a result, the model's performance can be reduced. After eliminating texts from the images, some specific lines, blobs, and noise are still visible. Removing these effectively is a crucial step in pre-processing images. Morphological opening is applied to remove these kinds of artifacts. In morphological opening, erosion is applied to eliminate small blobs, followed by dilation to regrow the original object [72]. It requires two inputs, the original image as well as a second input known as a structural element or kernel to determine the operation procedure. The cv2.getStructuringElement function creates a rectangular kernel (5, 5) to extract straight, vertical, or horizontal lines and noises. The size of the kernel or filter relies on the operations performed. The resulting output is shown in Fig. 7.

#### 3.3.4 Removing speckle noise

The visual quality of ultrasound images, which are low-resolution images generated by reflecting ultrasonic waves, is impacted by various types of noise, such as speckle noise [78]. Speckle noise may negatively influence image segmentation and compression at the post-processing stage [75]. Speckle noise is the association of additive and multiplicative noise that is statistically independent of the original image [79]. The expression of an image with speckle noise [80] is given below (equation (1)):

(1) g(p,q)=f(p,q)*u(p,q)+η(p,q)

where, g(p, q) is the tainted image; u(p, q) denotes multiplicative noise; f(p,q) is original image and ***η***(p,q) is additive noise.

De-noising ultrasound images before diagnosis is important because speckle noise impairs image quality. Speckle noise has been eliminated from medical ultrasound images using various filtering techniques [81]. Here a median filter is used to remove speckle. Median filtering is one of the best-known techniques to remove speckle noise from images [75]. Conventional smoothing filters remove noise from the input but cannot preserve the edges of the signal. In contrast, a median filter is a unique smoothing filter that enhances the outcome by eliminating noise from the signal while preserving the edges [81]. Edge preservation is an essential property because edges are important for visual appearance. The central element is exchanged with the median value acquired from the cv.medianBlur() function that takes median of all the pixels under the kernel area.

Table 4 shows which algorithms, functions and parameter values are used for preprocessing. The Text Removal process uses the Image Inpainting algorithm with the “cv2.INPAINT_NS” function in the OpenCV library to remove text from the images. Artifact Removal uses the Morphological opening algorithm with the “cv2.morphologyEx()" function and a structuring element of rectangular shape and kernel size (5,5). Speckle noise removal uses Median filtering with the “cv2.medianBlur()" function and a kernel size of (5), (5). Image resizing can be performed with the “cv2.imresize()" function.

**Table 4:** Pre-processing algorithms.

Process	Algorithm	Function/Method	Parameters
Text Removal	Image Inpainting	cv2.INPAINT_NS ()	
	Bitwise and		
Artifact Removal	Morphological opening	cv2.getStructuringElement()	Structuring element = cv2.MORPH_RECT, kernel Size = (5,5)
		cv2.morphologyEx()	Morphological operation = cv2.MORPH_OPEN
Speckle noise Removal	Median filtering	cv2.medianBlur()	Kernel Size = (5,5)
Image resizes		cv2.imresize()	

### 3.4 Verification

Numerous methods have been utilized for image pre-processing, some of which may impact image condition. Statistical analysis is used to make sure the image quality is not diminished. This study calculates the PSNR, MSE, SSIM, and RMSE values to analyze image quality.

#### 3.4.1 MSE

The second instance of the error function compared to the original and reconstructed images is known as the MSE [1]. MSE defines the difference between two images beneath comparison as the cumulative squared error of the pixel-to-pixel difference. The MSE value ranges from 0 to 1, with a value close to 0 representing acceptable image quality and a value of 0 indicating an image with no disturbance. Values greater than 0.5 signify a drop in quality [82,83]. The expression for Mean Squared Error (MSE) is typically provide as in equation (2):

(2) $$\text{MSE}=\frac{1}{\text{mn}}\sum_{i=0}^{m-1}\sum_{j=0}^{n-1}{\left(O\left(i,j\right)-P\left(i,j\right)\right)}^{2}$$

where, O represents the original image, P represents the pre-processed image, *I* and *j* are the row and column indices of the pixels of O and P, and m and n are the number of rows and columns respectively.

#### 3.4.2 PSNR

PSNR Ratio measures the proportion of the highest signal power and the noise [75,84]. This ratio can be used to compare the original and the processed images' quality. Equation (3) expresses PSNR mathematically:

(3) $$PSNR=10\;\log_{10}\left(\frac{R^{2}}{MSE}\right)$$

R exhibits the highest variation in the input image data.

#### 3.4.3 RMSE

The root mean square error (RMSE) is also known as root means square deviation (RMSD) [85] and calculates the quality difference between the original and pre-processed images. Low RMSE values, particularly those near 0, indicate less error and high image quality [74]. Equation (4) expresses RMSE:

(4) $$RMSE=\sqrt{\sum_{j=1}^{N}\frac{d_{fi}-{d_{d}}^{2}}{N}}$$

where, $d_{fi}$ stands for the predicted value; $d_{d}$ stands for actual value and N stands for size of the Dataset.

#### 3.4.4 SSIM

SSIM can be used as a measure of the impact or reduction on image quality of preprocessing measure. The value of the SSIM ranges from −1 to 1, where 1 represents perfect structural similarity and 0 denotes no similarity [30]. Equation (5) express mathematical interpretation of SSIM:

(5) $$SSIM\left(x,y\right)=\frac{\left(2\mu_{x}\mu_{y}+c_{1}\right)\;\left(2\sigma_{xy}+c_{2}\right)}{\left(\mu_{x}^{2}+\mu_{x}^{2}+c_{1}\right)\;\left(\sigma_{x}^{2}+\sigma_{x}^{2}+c_{2}\right)}$$

where, x, y represents the two images, $\sigma_{x}^{2},\sigma_{y}^{2}$ the variance and $\sigma_{xy}$ the covariance of the images; $\mu_{x},\mu_{y}$ are two images average compute with the Gaussian window and; $c_{1},c_{2}$ are two factors to maintain the division. In Table 5 shows these statistical values of 20 images.

**Table 5:** Statistical values for 20 images to compare image quality.

Image	MSE	PSNR	SSIM	RMSE
Image_1	15.17	39.35	0.962	0.13
Image_2	13.63	40.66	0.961	0.13
Image_3	14.25	42.59	0.964	0.11
Image_4	13.13	38.28	0.968	0.12
Image_5	12.38	45.47	0.962	0.09
Image_6	13.69	35.65	0.967	0.10
Image_7	13.98	39.28	0.966	0.11
Image_8	13.77	36.41	0.973	0.09
Image_9	15.09	46.22	0.931	0.12
Image_10	15.47	40.25	0.964	0.09
Image_11	12.19	41.79	0.968	0.10
Image_12	12.54	39.31	0.962	0.11
Image_13	12.47	42.45	0.966	0.09
Image_14	15.22	42.63	0.973	0.12
Image_15	12.34	46.22	0.931	0.09
Image_16	12.39	40.25	0.964	0.10
Image_17	14.99	42.45	0.968	0.11
Image_18	14.52	42.63	0.987	0.12
Image_19	14.63	46.22	0.948	0.10
Image_20	14.39	40.25	0.996	0.11

A total of 20 pre-processed images are presented in Table 5, which shows that the quality of pre-processed images remains uncompromised across the entire dataset, with essential feature information effectively preserved after a large amount of processing.

### 3.5 Proposed segmentation and classification approach

In this study, a CAD system has been constructed to utilize breast ultrasound images to segment and classify breast cancer. A UNet model is used for segmentation and a CNN model for classification using the segmented dataset. An ablation study is conducted to determine the optimal configuration. The RKO-UNet network is developed for segmentation by performing two architectural experiments and six hyperparameter experiments of the UNet model. The RKONet-13 model is developed for classification using nine hyperparameter experiments and one architectural experiment. Section 4.2 shows the detailed description of the findings of the ablation study.

#### 3.5.1 Proposed segmentation model

Image segmentation is an essential process in computer vision [86], like image processing, separating an image into multiple meaningful parts [87]. In medical imaging, segmentation can help to extract useful information, aiding in treatment planning and diagnosis [86]. UNet is a Special type of architecture for image semantic segmentation. This study builds on a base UNet-like architecture which is developed from scratch. The optimal network is determined through ablation study. During the training phase, encoder, decoder, filter size, kernel_Initilizer, activation function, Dropout, optimizer, learning rate, and batch normalization are modified and updated to get the best and most robust model.

#### 3.5.2 Base UNet

**Fig. 8:**
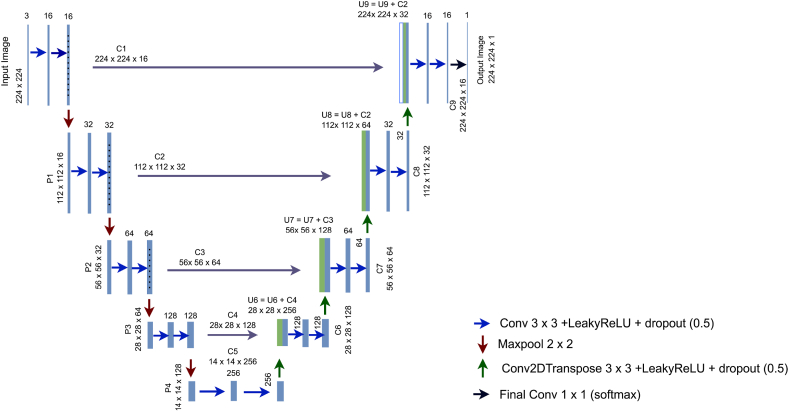
Base UNet like architecture.

The base U-Net architecture is comprised of an encoder located on the left and a symmetric decoder situated on the right. The encoder and the decoder repeat two convolutional layers with the same padding, feature dimension (filter size) of 16, and 3 X3 convolutional kernels. The encoder uses downsampling with maxpool to decrease the feature map's spatial dimension and then increase the number of feature channels. On the other hand, the decoder uses the deconvolution layer for up-sampling, to restore the feature details. Low-level and high-level feature maps are combined in a cross-layer. LeakyReLU is relied on as the activation function, and sigmoid is used as the activation for the last layer. Binary_crossentropy is used as the loss function with the ‘Adam’ optimizer and a dropout of 0.1. The kernel initializer is ‘he_normal’ and the learning rate is 0.001. Initially, three encoders and three decoders are used in the base UNet like architecture. Fig. 8 shows the fundamental architecture of the baseline UNet.

#### 3.5.3 Training approach

To train the baseline UNet architecture, initially the highest number of epochs is set to 200 with Adam as the optimizer and a learning rate of 0.001. ‘Binary cross-entropy is employed as the default loss function and ‘sigmoid’ for activation of semantic segmentation. The sigmoid function always works for binary (semantic segmentation) segmentation and is mainly used for models that predict the probability. The output of the sigmoid function ranges from 0 to 1. Sigmoid can be mathematically expressed as equation (6):

(6) $$sigmoid=\frac{1}{1+e^{-X}}$$

where, X is the Input and e is Euler's number.

All the experiments of this study were done on a computer with an Intel Core i5-10400F processor, NVidia GeForce GTX 1080 GPU, 16 GB of primary memory and Samsung 980 Pro 500 GB PCIe 4.0 M.2 NVMe SSD for storage.

#### 3.5.4 RKO-UNet

**Fig. 9:**
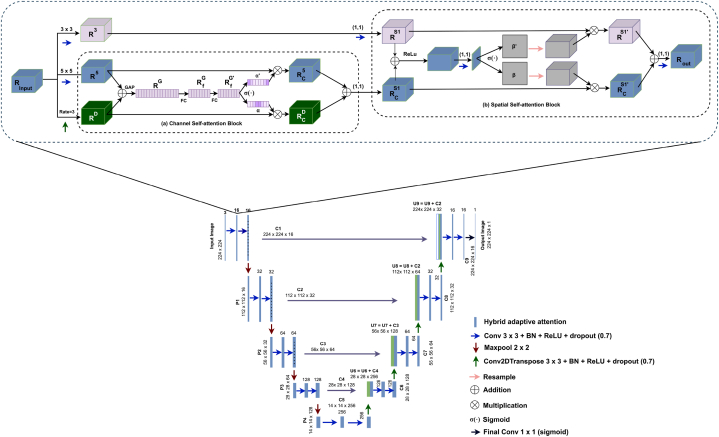
Hybrid attention UNet (RKO-UNet).

A shallow architecture is proposed for image segmentation with several modules and layers, including input, convolutional, batch normalization, activation, dilated rate, hybrid attention module, pooling, dropout, and output layers. An ablation study is performed to find out the optimal layer architecture and configuration of a UNet model based on two architectural experiments and six hyperparameter experiments. A detailed description of the findings of this ablation study can be found in section 4.2.1. In Fig. 9 depicted the RKO-UNet model structure of this study.

The proposed hybrid attention UNet (RKO-UNet) has four down-sampling, four up-sampling and four skip connections similar to UNet [88]. Every encoding and decoding stage have two hybrid attention modules (a channel self-attention block and a spatial self-attention block). Binary crossentropy, which measures the difference between anticipated masks and actual values [88], is used as the loss function. The proposed hybrid attention unit comprises three concurrent convolutional layers (one 3 x 3, one 5 x 5 and one 3 x 3 dilated convolution with dilation rate 3), channel self-attention and spatial self-attention block. It can be mathematically expressed as equations (7), (8), (9):

(7) $$R^{3}=W_{3\times 3}\times R_{input}$$

(8) $$R^{5}=W_{5\times 5}∙R_{input}$$

(9) $$R^{D}=W_{3\times 3}^{D}∙R_{input}$$

Here, $R_{input}\in R^{c^{\prime }\times h\times w}$ ; $R^{3}\in R^{c\times h\times w}$; $R^{D}\in R^{c\times h\times w}$.where, $W_{3\times 3}$ and $W_{5\times 5}$ is the Matrix of a 3 x 3 and 5 x 5 convolution and $W_{3\times 3}^{D}$ is the Matrix of a 3 x 3 dilated convolution.

##### 3.5.4.1 Channel self attention

The purpose of the Channel Self-attention block is to assist the segmentation network to select more feature representations and concentrate on the feature's category [89]. The channel self-attention block is depicted in Fig. 9. It utilizes a global average pooling (GAP) technique to quickly integrate the merged feature maps of $R^{5}\in R^{c\times h\times w}$ and $R^{D}\in R^{c\times h\times w}$ into a new feature map $R^{G}\in R^{2c\times 1\times 1}$ of size 1x1. The generated feature map can be described as follows equation (10):

(10) $$R^{G}=GAP\left(R^{5}⊕R^{D}\right)$$

where, $R^{5}$ is the feature map obtained from a 5 x 5 convolution layer, $R^{D}$ is the feature map from the dilated convolution layer; ⊕ denotes elementwise adding together and, $R^{G}$ is the new feature map.

To create a new feature, $R^{G}$ is fed into a fully connected layer, afterwards a batch-normalization layer, ReLU, and a dropout of 0.7. The newly developed feature map can be described as follows equation (11):

(11) $$R_{f}^{G}=\sigma_{r}\left(B\left(W_{fc}∙R^{G}\right)\right)$$

where, $W_{fc}$ is the matrix of fully connected layers; B is batch-normalization, $\sigma_{r}$ is ReLU activation, $R^{G}$ is the input feature map, and $R_{f}^{G}$ is the new feature map.

$R_{f}^{G}$ is the input of another fully connected operation resulting in a newly updated feature map. equation (12) express the new feature map in the bellow:

(12) $$R_{f}^{G^{\prime }}=W_{fc}∙R_{f}^{G}$$

where, $W_{fc}$ is the matrix of fully connected layers; $R_{f}^{G}$ is previous feature map, used as an input, and $R_{f}^{G^{\prime }}$ is the output (new feature map).

The new feature map $R_{f}^{G^{\prime }}$ is used as an input for the sigmoid activation to produce a channel attention map and equation (13) express it in mathematically:

(13) $$\alpha =\sigma_{s}\left(R_{f}^{G^{\prime }}\right)$$

Let, $\alpha \in {\left[0,1\right]}^{c\times h\times w}$ and $\alpha^{\prime }\in {\left[0,1\right]}^{c\times h\times w}$.where, α= channel attention maps of $R^{D}$ and $\alpha^{\prime }=$ channel attention maps of $R^{3}$.

Each value of $\frac{\alpha }{\alpha^{\prime }}$ represents the significance of the channel information at the associated voxel in $\frac{R^{D}}{R^{5}}$. The α and $\alpha^{\prime }$ Channel Attention Maps assist in adaptive extracting of more relevant feature maps from receptive fields with various scales. α calibrates with $R^{D}$, and α’ calibrates with D for automatic feature selection, and the expression can be written as below equations (14), (15)):

(14) $$R_{C}^{D}=\alpha ⊗R^{D}$$

(15) $$R_{C}^{5}=\alpha^{\prime }⊗R^{5}$$

Here, $R_{C}^{D}\in R^{c\times h\times w}$ and $R_{C}^{5}\in R^{c\times h\times w}$.

$R_{C}^{D}$ and $R_{C}^{5}$ feature maps are combined and utilized as the input for the following phase.

##### 3.5.4.2 Spatial self-attention block

Spatial attention targets feature location and placement [90]. To enhance the performance of the model, a spatial self-attention block is created, see Fig. 9. The channel self-attention block is employed as input to a 3 x 3 convolutional layer. A 1 x 1 convolution operation is conducted on the input feature map, to improve the target's exact location information. The mathematical expression 16 and 17 is given below:

(16) $$R^{S1}=W_{1\times 1}∙R^{3}$$

(17) $$R_{C}^{S1}=W_{1\times 1}∙\left(R_{C}^{5}⊕R_{C}^{D}\right)$$

where $R_{C}^{5},R_{C}^{D}$ is the input (Output of the channel self-attention block) and $R^{3}$ is the feature map of 3 x 2 convolution.

The extracted features fused with F and G with a ReLU activation function and a 1 × 1 convolution with a sigmoid activation to create the spatial attention mapping. The equation can be expressed as follows (equation (18)):

(18) $$\beta =\sigma (W_{1\times 1}∙\sigma_{r}\left(R^{S1}⊕R_{C}^{S1}\right)$$

where, $R^{S1},R_{C}^{S1}$ represent the input, ⊕ stands for element-wise addition, β is a spatial attention map of $R_{C}^{S1}$ and $\beta^{\prime }$ is a spatial attention map of $R^{S1}$.

Let, β∈[0,1] and $\beta^{\prime }\in \left[0,1\right]$. Each value of $\frac{\beta }{\beta^{\prime }}$ denotes the implication of the channel information at the associated voxel in $\frac{R_{C}^{S1}}{R^{S1}}$. A spatial attention map with the same number of channels as $R_{C}^{S1}$ is created by resampling to calibrate β. The resampling process is carried out similarly for $\beta^{\prime }$. The feature maps scaled by β and $\beta^{\prime }$ can be defined as $R_{C}^{{S1}^{\prime }}$ and $R^{{S1}^{\prime }}$, respectively. The output of the spatial self-attention block is produced by performing a convolution operation on the associated $R_{C}^{{S1}^{\prime }}$ and $R^{{S1}^{\prime }}$. Equation (19) provide the expression:

(19) $$R_{out}=W_{1\times 1}∙\left(R_{C}^{{S1}^{\prime }}⊕R^{{S1}^{\prime }}\right)$$

where $R_{out}$ is the output of the whole hybrid attention module; ⊕ is element-wise addition.

##### 3.5.4.3 Statistical analysis to evaluate the segmentation performance

Immediately following performing ROI segmentation using the proposed model, various statistical metrics are used to assess its performance. The segmentation performance is evaluated by comparing the segmented images with the corresponding ground truth image that comes with the dataset. These include PSNR, Dice Similarity Score, Normalized Cross Correlation, Normalized Mutual Information, Sum of Squared Differences, RMSE, Mean Squared Error, Feature Based Similarity Index, and Universal Image Quality Index. These metrics provide a comprehensive evaluation of the segmentation results and help evaluate the efficiency of the proposed model in accurately identifying and separating the ROI from the rest of the image.

##### 3.5.4.4 Dice Similarity Score (DSC)

The DSC is the primary validation metric for the spatial overlap index [91] and evaluates the pixel-level consistency between a predicted segmentation and the associated ground truth [92]. Equation (20) provides the expression for DSC:

(20) $${DSC}_{P,Q}=\frac{2\left(P⋂Q\right)}{\left|P\right|+\left|Q\right|}$$

where, P is the segmented image, Q is the corresponding ground truth and ${DSC}_{P,Q}$ is the Dice Score. When the dice similarity coefficient value is one, the segmented mask and ground truth overlap entirely, and the segmentation is good. The range of the dice coefficient value is 0–1 [93].

##### 3.5.4.5 Sum of squared difference (SSD)

The sum of squared differences is a measure of similarity, depends on the pixel-by-pixel intensity differences between two images [94,95]. It is the summation of squares pixels subtraction between two images [95,96]. This can be expressed as follows (equation (21)):

(21) $${SSD}_{\left(i,j\right)}=\sum_{i=0}^{X}\sum_{j=0}^{Y}{\left(P\left(i,j\right)-Q\left(i+a,j+b\right)\right)}^{2}$$

where P is first image, Q is the second image, X is the reference image row size; Y is the reference image column size; a is the shift component of x-axis, and b is the shift component of y-axis. The values of a and b depend on the specific application and the degree of misalignment between the two images. The values of a and b were set to zero in this study.

#### 3.5.5 FSIM

FSIM [97,98] (Feature-Based Similarity Index) is an image quality evaluation metric which assesses similarities between two images depending on their structural and perceptual features. The metric is defined as a ratio of joint statistics of two feature vectors. It uses a set of predefined filters to extract structural and perceptual features, allowing it to provide a more accurate measure of similarity than pixel-based metrics.

##### 3.5.5.1 Universal image quality index

A universal image quality index combines loss of correlation, luminance distortion, and contrast distortion to characterize how an image deviates from a reference image [98].

##### 3.5.5.2 Jaccard similarity index

The Jaccard similarity index is also known as the Jaccard similarity coefficient. It calculates the similarity between two sets of data, ranging from 0 to 1. A high value specifies a high degree of similarity, while a low value denotes a low degree of similarity. Jaccard similarity index is expressed by equation (22):

(22) $$J\left(P,Q\right)=\frac{|P⋂Q|}{\left|P⋃Q\right|}$$

Where, J = Jaccard distance; P = data(image) set 1; Q = data(image) set 2.

##### 3.5.5.3 Normalized Mutual Information

Normalized Mutual Information (NMI) is a determine of the similarity between two images. It is commonly used in image segmentation and information retrieval to evaluate the results. The NMI value ranges between 0 and 1, where a higher value indicates a better match between the two images [99,100].

### 3.6 Proposed classification model

A base CNN model is generated. The Convolution layer, Maxpool layer, filter size, number of filters, pooling layer, activation function, batch size, flatten layer, and loss function are then modified to get the best performing model. The model is trained with the segmented dataset which has been split into training, testing and validation sets.

#### 3.6.1 Split dataset

**Fig. 10:**
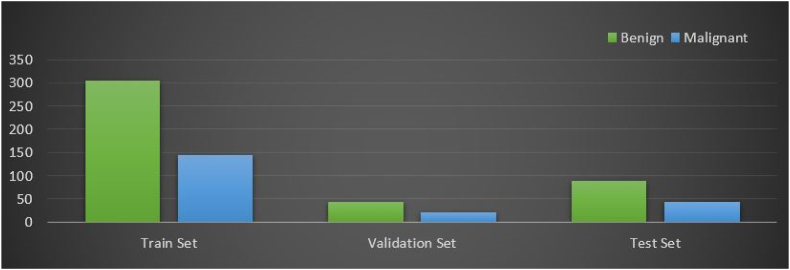
Class distribution of training, validation, and test sets after splitting the segmentation dataset.

After getting the ROIs, the segmented dataset is split before training the classification model. In this study, 70 % of the data is used for model training, 20 % for testing, and 10 % for validation [101]. Since validation data is evaluated at each epoch during the training stage, the computational time of epochs will increase for a more extensive validation dataset. The training, testing, and Validation sets consist of 302, 201, and 100 images, respectively. Fig. 10 shows the distribution of ultrasound images for the two classes in the training, testing, and validation sets.

#### 3.6.2 Base CNN model

**Fig. 11:**
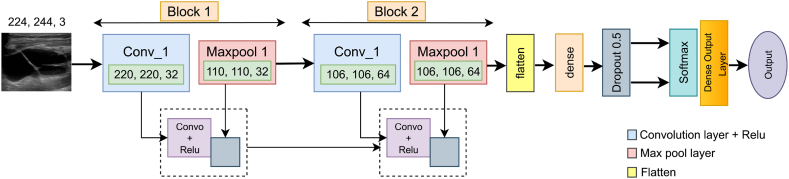
Base CNN model.

A CNN model with two convolutional layers, each followed by a maxpool layer is used as the base model. Initially, the model has 3 X 3 convolutional kernels. In the first block, the convolutional kernel has 32 and 64 for the second block, with a dropout value of 0.5. The selected activation function is ‘ReLu,’ ‘SoftMax’ is the final layer activation function. ‘categorical crossentropy’ is used as the loss function with batch size 32 and the ‘Nadam’ optimizer. The model runs for 100 iterations on the segmented dataset with the 224 x 224 x 3 input dimensions. The learning rate is set to 0.001. The feature map in a convolutional layer is generated by dot operation of the input and weight. The baseline model of this study is shown below in Fig. 11.

The base model is show below.

#### 3.6.3 Training approach

The utmost amount of training epochs was set to 100, the batch size to 16, with the Adam optimizer and a learning rate of 0.001 to train the models. The loss function is “categorical cross-entropy.” ‘Softmax’ activation is applied to predict the possibility for each class. Because Softmax normalizes each value between 0 and 1, their aggregate always equals 1. If the probabilities of one class change, the probabilities of the other classes also change, preventing a total change in probabilities.

#### 3.6.4 RkoNet-13

**Fig. 12:**
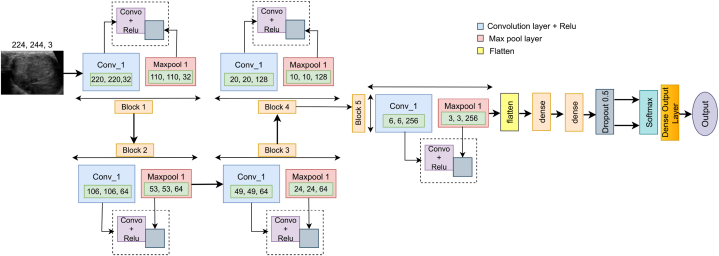
RkoNet-13 model.

This study optimizes the base architecture with various modules and layers, including input, convolutional, activation, pooling, fully connected, dropout, and dense output layers. Fig. 12 shows the robust CNN (RKONet-13) model which got after doing architectural and hyperparameter ablation study.

The proposed RKONet-13 has 13 layers, encompassing five convolutional, five max-pooling, and three dense layers. The model has thirteen layers. This model architecture has five blocks, each with 3 x 3 convolutional layers with ReLU activation, 1 x 1 stride size, and a 2 x 2 max-pooling layer. RkoNet-13 has a total of 4,592,514 trainable parameters. Throughout the model training, preliminary weights retrieve attributes from the input data, and the loss function determines the architecture error rate. After each training epoch, kernel weights are altered depending on the error rate.

Block-1's initial convolutional layer comprises 32 filters and 2432 trainable parameters. Block-1's convolutional layer has 32 filters that retain structural features and textural qualities of input ultrasound images. This creates 32 feature maps for each input, which are then corrected using ReLU to maintain only non-negative values. The first convolutional layer's output feature maps are then reduced by half utilizing a 2 x 2 max-pool layer. After that, the feature maps are passed as input data to Block 2.

Blocks 2 and 3 each include a convolutional layer of 64 filters with 5 x 5 kernel size, and the number of trainable parameters are 51264 and 102464, respectively, with the ReLU activation function. A 2 x 2 max-pool layer follows every convolutional layer of Block-2 and Block-3, and two blocks shrink the feature maps created by Block 1 to half their original size. The reduced feature map is used as an input of block 4.

Block-4 has a convolutional layer with 5- × -5-kernel-size with 128 filters, and the total trainable parameters is 204928. A 2- × −2 max-pooling layer reduces the feature map to half, and creates the input of the 5th block.

Block-5 has 5 × 5 convolutional layers, including 256 filters and the total trainable parameters are 819456 with a 2 × 2 max-pooling layer.

### 3.7 Transfer learning models

The proposed classification model was compared with five well-known transfer models: VGG16, VGG19, ResNet50, alexNet, and InceptionV3.

#### 3.7.1 VGG16

The VGG16 architecture is a convolutional neural network with a depth of 16 layers, and it employs a 3 x 3 receptive field and six max pool layers. The most distinctive feature of VGG16 is using 3x3 convolution layers with stride 1 and constantly using 2x2 padding and maxpool layers. Convolution and max pool layers are organized in a similar way in the entire design. Softmax is used, pursued by two fully connected layers (FC).

#### 3.7.2 VGG19

VGG19 is a convolutional neural network with a depth of 19 layers. It employs a 3 x 3 receptive field, sixteen convolution layers, five max pool layers, three fully connected layers, and one softmax layer. The convolution layers have 3x3 filters with a stride one and constantly using the spatial padding and maxpool layer of 2x2 filters with a stride 2. Ubique the design, the convolution and max pool layers are positioned in the similar manner. The first two connected layers' sizes are 4096, and since that, a layer with 1000 channels for 1000-way ILSVRC classification and a softmax layer is the final layer.

#### 3.7.3 ResNet50

ResNet50 consists of 48 convolutional layers as well as maxpool layer and an average pool layer. The benefits of ResNet50 are that the large number of trainable parameters enables a very good performance.

#### 3.7.4 InceptionV3

The primary goal of InceptionV3 is to decrease the amount of computational power. It focuses on smaller convolutions, factorized convolutions, asymmetric convolutions, and grid size reduction. Inception V3 uses like label smoothing, it factorizes 7x7 convolutions and auxiliary classifiers are used to generate the output. This training time is relatively short.

#### 3.7.5 AlexNet

AlexNet is a deep convolutional neural network (CNN) that was proposed in 2012 and is widely considered as a pioneer in deep learning for computer vision. It was designed for the ImageNet Large Scale Visual Recognition Challenge and won the first place in the image classification task, outperforming previous state-of-the-art models by a large margin. AlexNet introduced several novel architectural features such as the rectified linear units (ReLU) activation function, dropout regularization, and data augmentation techniques, which have become standard components in modern CNNs.

## 4 Results

This section discusses the results, including an ablation study of the UNet and CNN models, a statistical analysis of the segmentation and classification models, a confusion matrix, loss and accuracy curves and, a comparison of the proposed model with transfer learning models using raw, pre-processed and segmented datasets. K-fold cross-validation is also done.

### 4.1 Evaluation metrics

This study used several metrics to assess the CNN and Transfer learning models: Precision, Recall, F1-score, accuracy (ACC), sensitivity, and specificity. For each model, a confusion matrix was yielded, and the values for true positive (TP), true negative (TN), false positive (FP), and false negative (FN) were obtained. The area under the curve (AUC) value was also computed. In addition, the false positive rate (FPR), false negative rate (FNR), false discovery rate (FDR), mean absolute error (MAE), and root mean squared error (RMSE) were computed. The true positive rate (TPR), also called Recall, and false positive rate (FPR) are plotted against each other at various threshold levels to produce the AUC value. Equation (23), (24), (25), (26), (27), (28), (29), (30), (31), (32), (33)) provides mathematical expressions:

(23) $$ACC=\frac{TP+TN}{TP+TN+FP+FN}$$

(24) $$\text{Recall}=\frac{\text{TP}}{\text{TP}+\text{FN}}$$

(25) $$\text{Specificity}=\frac{\text{TN}}{\text{TN}+\text{FP}}$$

(26) $$Precision=\frac{TP}{TP+FP}$$

(27) $$ACC=2\frac{precision*recall}{precision+recall}$$

(28) $$FPR=\frac{FP}{FP+TN}$$

(29) $$FNR=\frac{FN}{FN+TP}$$

(30) $$FDR=\frac{FP}{TP+FP}$$

(31) FPR=1−Specificity

(32) $$MAE=\frac{1}{n}\sum_{j=1}^{n}{\left(y_{j}-y_{j}^{p}\right)}^{2}$$

(33) $$RMSE=\sqrt{\frac{1}{n}\sum_{j=1}^{n}{\left(y_{j}-y_{j}^{p}\right)}^{2}}$$

### 4.2 Results of ablation study

In this section, the results of each experiment of architectural and hyper-parameter ablations are presented and discussed in detail, with a focus on the UNet and CNN models. These experiments were designed to evaluate the impact of various design choices on the performance of the models.

#### 4.2.1 Ablation study of UNet

The base UNet model achieves 88.94 % test accuracy for image segmentation. Eight experiments are performed to modify components of UNet, such as Encoder-Decoder, Activation, kernel Initializer, Batch Normalization, Optimizer, learning rate, and Dropout in order to get a robust model that achieves a high accuracy. The experimental outcomes are explained in Table 6.

**Table 6:** Ablation study for UNet with eight parameters.

Study 01: **Encoders and Decoders**				
**Configuration No.**	No. of encoders	No. of decoders	Test accuracy (%)	Finding
**1**	2	2	82.52	Lowest accuracy
**2**	3	3	88.23	intermediate accuracy
3	**4**	**4**	**88.94**	**Highest accuracy**
**4**	5	5	88.90	Intermediate accuracy
Study 2: **Changing Model Architecture by adding attention module**				
**Configuration No.**	Model Architecture		Test accuracy (%)	Finding
**1**	UNet		88.94	Previous and lowest accuracy
**2**	UNet with channel self-Attention block		89.23	Modest improvement
**3**	UNet with spatial self-Attention block		89.44	Modest improvement
4	**UNet with Hybrid attention block**		**96.94**	**Highest accuracy**
Study 3: **Activation function**				
**Configuration No.**	Activation		Test accuracy (%)	Finding
**1**	PReLU		92.78	accuracy deterioration
2	**ReLU**		**97.94**	**Highest accuracy**
**3**	Leaky ReLU		96.94	previous accuracy
**4**	Tanh		89.27	accuracy deterioration
**5**	Elu		83.29	accuracy deterioration
Study 4: **Kernel Initializer**				
**Configuration No.**	Kernel Initializer		Test accuracy (%)	Finding
**1**	Initializer		96.65	accuracy deterioration
**2**	normal		96.77	accuracy deterioration
3	**he_normal**		**97.94**	**Highest accuracy**
**4**	identity		96.34	accuracy deterioration
**5**	constant		95.59	accuracy deterioration
Study 5: **Batch Normalization**				
**Configuration No.**	Batch Normalization		Test accuracy (%)	Finding
**1**	Without Batch Normalization		97.94	previous and lowest accuracy
2	**With Batch Normalization**		**98.98**	**Highest accuracy**
Study 6: **Optimizer**				
**Configuration No.**	Optimizer		Test accuracy (%)	Finding
1	**Adam**		**98.98**	**Highest accuracy**
**2**	Nadam		95.98	accuracy deterioration
**3**	Adamax		95.45	accuracy deterioration
**4**	SGD		92.68	accuracy deterioration
**5**	RMSprop		90.82	Lowest accuracy
Study 7: **learning rate**				
**Configuration No.**	Leaning Rate		Test accuracy (%)	Finding
1	**0.01**		**99.01**	**Highest accuracy**
**2**	0.001		98.98	Previous accuracy
**3**	0.0001		97.67	accuracy deterioration
**4**	0.07		97.91	accuracy deterioration
**5**	0.007		97.49	Lowest accuracy
Study 8: **Dropout**				
**Configuration No.**	Dropout		Test accuracy (%)	Finding
**1**	0.01		99.01	previous accuracy
**2**	0.03		97.59	accuracy deterioration
**3**	0.05		94.67	Lowest accuracy
4	**0.07**		**99.14**	**Highest accuracy**
**5**	0.09		97.31	accuracy deterioration

**Fig. 13:**
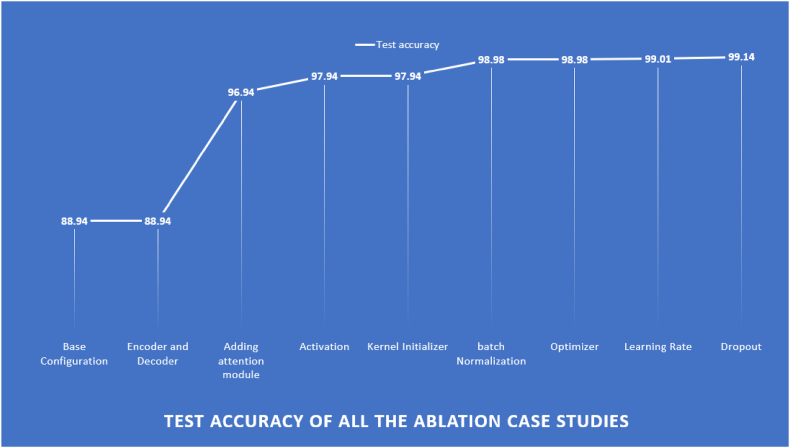
Test accuracy of all the ablation studies of UNet.

In Table 6, study 1, experiment with different numbers of encoders and decoders. As previously explained, encoders perform down-sampling and decrease spatial dimensions. On the other hand, decoders perform up-sampling to increase the feature's spatial dimension. The study consisted of four configurations with varying numbers of encoders and decoders. Configuration 3, with four encoders and decoders, achieved the highest accuracy of 88.94 %. The integration of an attention layer into the model enhances the model's ability to concentrate on the most important features. This can result in an improvement of the model's performance and an increase in its accuracy, contributing to a more effective system. In study 2, four different configurations of the UNet model were evaluated for their accuracy in detecting a certain finding. The baseline model achieved an accuracy of 88.94 %, while the addition of self-attention blocks resulted in modest improvements. However, the highest accuracy of 96.94 % is achieved with the UNet model using a Hybrid attention block. The activation function establishes whether to stimulate a neuron by producing a weighted sum and adding bias. It also involves learning and making sense of non-linear and complex mappings between the inputs and corresponding outputs [102]. Different activation functions can yield different accuracies. The third study shows experiments on various activation functions, PReLU, ReLU, Leaky ReLU, Tanh, and Elu, to find the best activation function in terms of accuracy. Initially, the activation function of the model was LeakyReLU, but ReLU produces the highest test accuracy of 97.94 %, and Elu yields the lowest accuracy. Kernel initializers may be used as the initial weights since it generates and distributes them [99,103]. In study 4, five kernel initializers, Initializer, normal, he_normal, identity, and constant, are employed, where he_normal performed best with a test accuracy of 97.94 %. Batch Normalization enables greater learning rates. Additionally, it serves as a regularizer, often removing the necessity for Dropout [104]. The experiments with and without Batch Normalization where Batch Normalization performed best with 98.98 % accuracy in study 5. An optimizer is a function that alters the weights and learning rates of the neural network's properties and refers to minimizing or maximizing the loss function [105]. Adam, Nadam, Adamax, SGD, and RMSprop were tried in study 6, where Adam provides the best test accuracy of98.98 %. The learning rate affects training speed and generalization accuracy [106]. The study 7 is experimented with learning rates of 0.01, 0.001, 0.0001, 0.07 where learning rate of 0.01 yielded the best test accuracy of 99.01 %. Dropout expands the performance of neural networks on supervised learning errands in vision and solves the overfitting problem [107]. The study 8 shows experiments with the dropout of 0.01, 0.03, 0.05, 0.07, and 0.09 and got the best test accuracy of 99.14 % with a dropout of 0.07. The increase of the test accuracies for each ablation studies on the UNet models are shown in Fig. 13.

As can be seen in Fig. 13, the base configuration of the model yielded an accuracy of 88.94 %. During the modification of the number of encoders and decoders, the highest achieved accuracy was 88.94 %. However, after adding an attention module, the accuracy increased to 96.94 %. Further improvement was seen when the activation function was changed, resulting in an accuracy of 97.94 %. The accuracy has further improved to 97.94 % after altering the kernel initializer. The addition of batch normalization and changing the optimizer resulted in an even higher accuracy of 98.98 %. After further modifications, the accuracy reached 99.01 %. Finally, after changing the dropout, the highest accuracy of 99.14 % was achieved.

#### 4.2.2 Ablation study of the CNN classification model

This section presents results from ten architectural and hyperparameter ablation studies performed on a base CNN model to create the robust RKONet-13 model. The base CNN model achieves 87.3 % test accuracy based on segmented ROI. The results in Table 7 show the impact of different configurations on the model's performance.

**Table 7:** Ablation study for optimizing the CNN model.

Study 1: Modifying the convolution layers and maxpool layers					
Configuration No.	No. of convolution layers	No. of pooling layers	Epoch x training time	Test accuracy (%)	Finding
1	2	2	97 × 8s	87.30 %	Lowest accuracy
2	3	3	95 × 7s	90.48 %	Intermediate accuracy
3	4	4	92 × 6s	93.24 %	Intermediate accuracy
**4**	**5**	**5**	**88 × 5s**	**93.83 %**	**Highest accuracy**
5	6	6	94 × 6s	92.00 %	Intermediate accuracy
**Study 2:** Modifying the filter size					
Configuration No.	Filter size	Epoch x training time	Test accuracy (%)	Finding	
1	2 x 2	88 × 5s	91.76 %	Lowest accuracy	
2	3 x 3	90 × 5s	93.83 %	Previous accuracy	
**3**	**5 x 5**	**85 × 5s**	**94.41 %**	**Highest accuracy**	
**Study 3:** Modifying the number of kernels					
Configuration No.	No. of kernels	Epoch x training time	Test accuracy (%)	Finding	
1	32 → 32→32 → 32→32	53 × 4s	93.65 %	accuracy deterioration	
2	32 → 32→32 → 64→64	39 × 5s	92.71 %	lowest accuracy	
3	16 → 32→64 → 128→256	65 × 4s	93.46 %	accuracy deterioration	
4	64 → 64→64 → 64→64	45 × 6s	94.24 %	accuracy deterioration	
5	64 → 64→64 → 128→256	65 × 5s	94.41 %	Modest accuracy	
**6**	**32 → 64→64 → 128→256**	**97 × 5s**	**95.08 %**	**Highest accuracy**	
**Study 4:** Modifying the pooling layer					
Configuration No.	Type of pooling layer	Epoch x training time	Test accuracy (%)	Finding	
**1**	**Max**	**97 × 5s**	**95.08 %**	**Highest accuracy**	
2	Average	61 × 6s	94.82 %	Accuracy deterioration	
**Study 5:** Modifying the activation function					
Configuration No.	Activation function	Epoch x training time	Test accuracy (%)	Finding	
1	PReLU	9 × 5s	93.65	accuracy deterioration	
**2**	**ReLu**	**97 × 5s**	**95.08 %**	**Highest accuracy**	
3	Leaky ReLu	88 × 5s	93.65 %	accuracy deterioration	
4	Tanh	5 × 5s	88.89 %	accuracy deterioration	
5	ELU	28 × 5s	88.88 %	accuracy deterioration	
6	Selu	25 × 5s	85.71 %	accuracy deterioration	
**Study 6:** Modifying the batch size					
Configuration No.	Batch size	Epoch x training time	Test accuracy (%)	Finding	
1	128	27 × 5s	92.06 %	accuracy deterioration	
2	64	82 × 5s	93.65 %	Accuracy deterioration	
3	32	43 × 4s	95.08 %	Previous accuracy	
**4**	**16**	**97 × 5s**	**96.93 %**	**Highest accuracy**	
**Study 7:** Modifying the Flatten Layer					
Configuration No.	Flatten layer type	Epoch x training time	Test accuracy (%)	Finding	
**1**	**Flatten**	**97 × 5s**	**96.93 %**	**Highest accuracy**	
2	Global Max pooling	60 × 4s	95.24 %	accuracy deterioration	
3	Global Average pooling	54 × 5s	96.83 %	accuracy deterioration	
**Study 8:** Modifying the Loss Function					
Configuration No.	Loss Function	Epoch x training time	Test accuracy (%)	Finding	
1	Binary Crossentropy	Error	Error	Error	
2	Mean squared logarithmic error	45 × 5s	96.83 %	accuracy deterioration	
3	Mean Squared Error	96 × 5s	96.82 %	accuracy deterioration	
**4**	**Categorical Crossentropy**	**97 × 5s**	**96.93 %**	**Highest accuracy**	
5	Mean absolute error	12 × 4s	68.25 %	accuracy deterioration	
**Study 9:** Modifying the Optimizer					
Configuration No.	Optimizer	Epoch x training time	Test accuracy (%)	Finding	
**1**	**Adam**	**97 × 5s**	**98.41 %**	**Highest accuracy**	
**2**	Adamax	88 × 5s	90.48 %	accuracy deterioration	
3	SGD	90 × 5s	84.13 %	accuracy deterioration	
4	Nadam	44 × 5s	96.93 %	Previous accuracy	
**Study 10:** Modifying the Learning Rate					
Configuration No.	Learning rate	Epoch x training time	Test accuracy (%)	Finding	
1	0.01	92 x 55s	91.46	accuracy deterioration	
**2**	**0.001**	**97 × 5s**	**98.41 %**	**Highest accuracy**	
3	0.0001	68 x 57s	97.28	accuracy deterioration	
4	0.007	87 x 56s	95.85	accuracy deterioration	
5	0.0007	65 x 57s	97.34	accuracy deterioration	

**Fig. 14:**
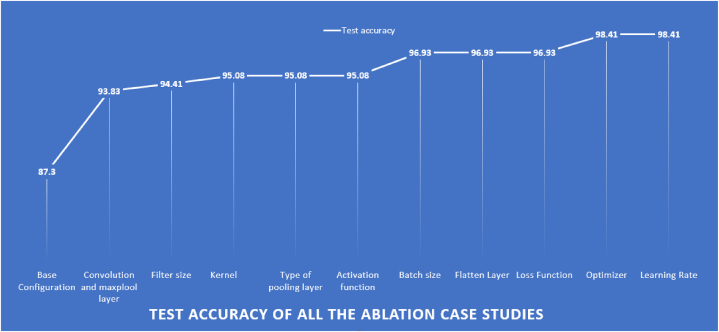
Test accuracy of all the ablation studies of CNN model.

The study 1 of Table 7 shows the models performance over different convolutional layers. A convolution layer performs feature extraction. Max-pooling accumulates patches from the input feature maps, outputs the largest value in each patch, and discards all other values [108]. Different numbers of convolutional and max-pool layers yield different accuracy. Initially, the model starts with two convolutional and two max-pool layers. After that, (3,3), (4,4), (5,5), (6,6), and (7,3) convolutional and max-pool layers were tried where five convolutional and five max-pool layers performed best with a 93.83 % test accuracy. The ideal filter size is often empirically determined to achieve the best performance and study 2 shows the experimental validation [109]. The experiment is done with several filter sizes like 3 x 3 pixels, 4 x 4 pixels, and 5 x 5 pixels, where 5 x 5 pixels yield the highest test accuracy, 94.41 %, with the lowest training time per epoch was 5 s. In study 3, several numbers of filters or kernels are experimented. Initially the number of filters is the same for all five convolutional layers (32, 32, 32, 32, 32). After that, the number of features is increased to 64, and the performance slightly improved. Different numbers are used in configuration 2,3,4,5, and 6. The highest accuracy, of 98.08 %, is achieved with configuration 6, where the number of filters for the five convolutional layers were 32, 64, 64, 128, and 256. The study 4 shows experiments with two types of pooling layers: max pool and average pool. The Max pooling layer achieves higher accuracy than the average pooling layer with a low epoch number. The Max pool and average pool layer resulted in 95.08 % and 94.82 % test accuracy, respectively. The study 5 has investigated ReLU, PReLU, Leaky ReLU, Tanh, Elu, and Selu to determine the most accurate activation function. The model's initial activation function was RelU, which produces a test accuracy of 95.08 %. The number of images utilized in a single forward and backward pass is called the batch size [110]. In study 6, an experiment is done with batch sizes of 16, 32, 64, and 128 and the highest accuracy is achieved with a batch size of 16 resulting in 96.93 % test accuracy. The flatten layer converts n-dimensional vector representations into 1-dimensional column vector representations for processing at the dense layer, transforming the feature map into a flattened output [111]. An experiment with different types of flatten layers is shown in study 7 where “Flatten” yields the highest accuracy, 96.93. In study 8, various loss functions, such as Binary Crossentropy, Categorical Crossentropy, Mean Squared Error, Mean Absolute Error, and Mean Squared Logarithmic Error are experimented and Categorical Crossentropy has performed best with a test accuracy of 96.93 %. The study 9 shows investigation on five optimizers: Adam, Nadam, Adamax, SGD, and RMSprop where Adam results in the highest test accuracy of 98.41 %. Finally, in study 10, several learning rates of 0.01, 0.007, 0.001, 0.0007, and 0.0001 are experimented. The best test accuracy was 98.41 % with a learning rate of 0.001. The increase of the accuracies for each ablation studies are shown in Fig. 14.

As demonstrated in Fig. 14, the base configuration model was initially able to accomplish an accuracy of 87.3 % on the test data. However, by enacting an ablation study, the accuracy of the model was improved to 98.41 %.

After the ablation study on the suggested RKONet-13 architecture, the classification accuracy has significantly increased and the training accuracy curve, validation accuracy curve, training loss curve across the training period, and confusion matrix were computed for the model. Table 8 provides an overview of the final configuration of RKONet-13.

**Table 8:** Configuration of proposed model RKONet-13.

Configuration	Value
Image size	224 x 224
Epochs	100
Optimization Function	Adam
Learning rate	0.001
Batch size	16
Activation function	Softmax
Dropout	0.5
Momentum	0.9

### 4.3 Analysis of the results of the best segmentation model

In this section, the results of the segmentation model are depicted in detail to provide an understanding of its accuracy and loss performance during training, testing, and validation.

**Fig. 15:**
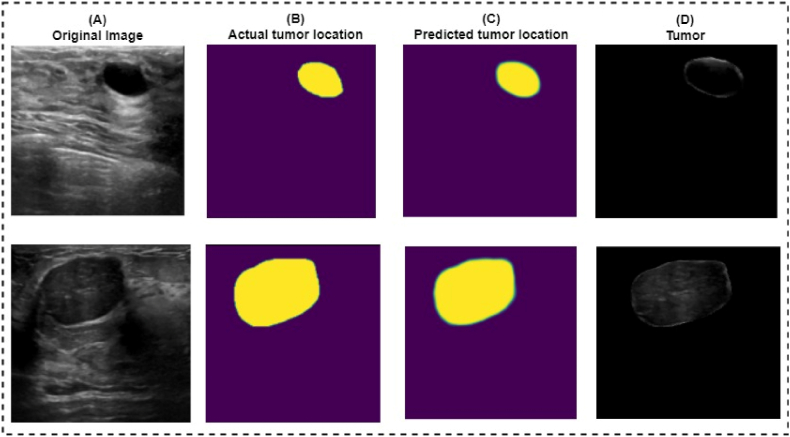
Similarity between actual and predicted location of tumor. (A) Original Images, (B) Actual tumor location, (C) Predicted tumor location. (D) Tumor.

An example of the output of segmentation is explained in Fig. 15. It can be seen that there is a close similarity between the actual location and the predicted location of the tumors.

Table 9 summarizes some of the outcomes of the proposed segmentation model. The model achieved 99.14 % test accuracy with 0.017 % test loss. The Dice similarity and Jaccard coefficient index of the model are 90.56 % and 91.33 %, respectively. The training and validation accuracy are 99.37 % and 99.14 %, respectively.

**Table 9:** RKO-UNet model performance.

RKO-UNet	Training Accuracy	99.37 %
	Training Loss	0.012
	Test Accuracy	99.14 %
	Test Loss	0.017
	Validation Accuracy	99.14 %
	Validation Loss	0.017
	Jaccard coefficient	91.33 %
	Dice Similarity	90.56 %

### 4.4 Statistical analysis

This analysis includes the evaluation of various metrics such as PSNR, SSIM, Dice Similarity [91], Normalized Cross Correlation, Normalized Mutual Information, Sum of Squared Differences [94,95], RMSE, MSE, Feature-Based Similarity Index [83,97] and Universal Image Quality Index [99]. These metrics have been used to quantify the similarity between the predicted and actual segmentations and to convey a comprehensive evaluation of the model's effectiveness.

Table 10 shows the performance metrics for 10 different images. But to get an overall view of the performance, we calculated the average values for each of these metrics across all images. The average PSNR was 47.97, indicating that the images have a high PSNR. The average SSIM was 0.989, showing that the images have a high level of structural similarity. The average Dice Similarity was 0.943, indicating that the images have a high level of overlap between their segmented regions. The average Normalized Cross Correlation was 0.954, which shows that the images have a high degree of correlation. The average Normalized Mutual Information was 0.965, which indicates that the images have a high level of shared information. The average Sum of Squared Difference was 0.23, indicating that the images have a low degree of difference. The average RMSE was 0.23, which shows that the images have a low degree of error. The average MSE was 0.05, indicating that the images have a low degree of average squared error. The average Feature-based Similarity Index was 0.957, indicating that the images have a high degree of similarity based on their features. Finally, the average Universal Image Quality Index was 0.965, showing that the images have a high level of quality.

**Table 10:** Segmentation results for ten different images.

Images	PSNR	SSIM	Dice Similarity	Normalized Cross Correlation	Normalized Mutual Information	Sum of squared difference	RMSE	MSE	Feature based similarity index	Universal image Quality index
Img1	46.81	0.99	0.96	0.95	0.97	0.1	0.1	0.5	0.97	0.98
Img2	45.79	0.98	0.95	0.93	0.95	0.2	0.2	0.6	0.97	0.97
Img3	46.29	0.99	0.93	0.95	0.96	0.1	0.1	0.5	0.93	0.94
Img4	49.21	0.99	0.95	0.96	0.98	0.2	0.1	0.4	0.97	0.98
Img5	48.96	0.98	0.92	0.93	0.94	0.4	0.3	0.8	0.95	0.95
Img6	49.29	0.99	0.93	0.97	0.98	0.2	0.2	0.4	0.96	0.97
Img7	46.37	0.98	0.96	0.98	0.99	0.4	0.3	0.6	0.97	0.98
Img8	46.29	0.99	0.93	0.95	0.96	0.4	0.3	0.8	0.93	0.94
Img9	49.21	0.99	0.95	0.96	0.98	0.2	0.1	0.4	0.97	0.98
Img10	49.29	0.99	0.93	0.97	0.98	0.2	0.2	0.4	0.96	0.97

### 4.5 Results for the classification RKONet-13 model

The following table shows some evaluation metrics for the best CNN model (RKONet-13): precision, recall, F1 score, specificity, sensitivity, training accuracy, training loss, test accuracy and test loss, and validation accuracy, validation loss.

There is a precision of 98.48 %, a recall of 1.00 %, an F1 score of 97.62, a specificity of 97.8 %, a sensitivity of 1.00 %, and a training accuracy of 98.98 % with corresponding test and validation accuracy of 98.98 %. Training, testing, and validation losses are 0.039, 0.304, and 0.304, respectively (see Table 11).

**Table 11:** Performance metrics of proposed model.

**Proposed Model**	Precision	98.48 %
	Recall	1.00 %
	F1 score	97.62 %
	Specificity	97.8 %
	Sensitivity	1.00 %
	Train Accuracy	98.98 %
	Train Loss	0.039
	Val Accuracy	98.41 %
	Val Loss	0.304
	Test Accuracy	98.41 %
	Test Loss	0.304

**Fig. 16:**
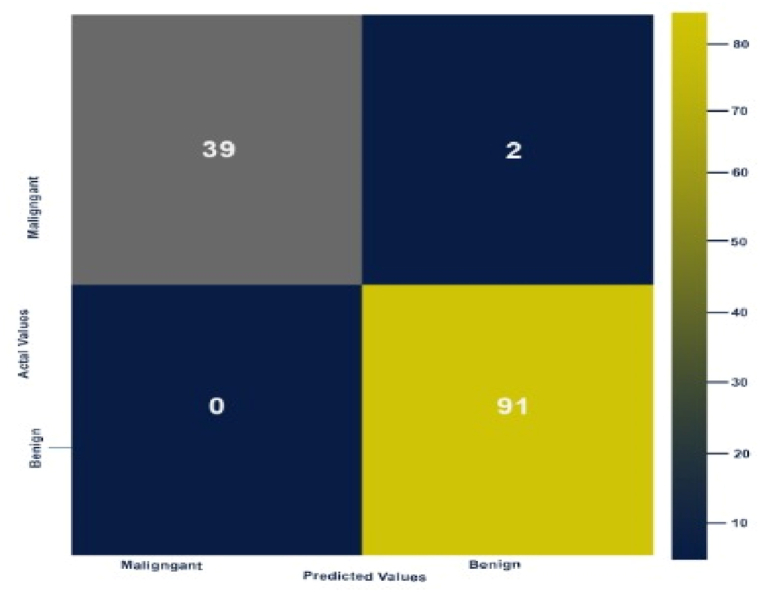
Confusion matrix of the proposed model.

The confusion matrix for the most accurate architecture is presented in Fig. 16. The column numbers indicate the predicted label, whereas the actual label of the test images is represented by the row values, and the diagonal values denote the True Positives.

**Fig. 17:**
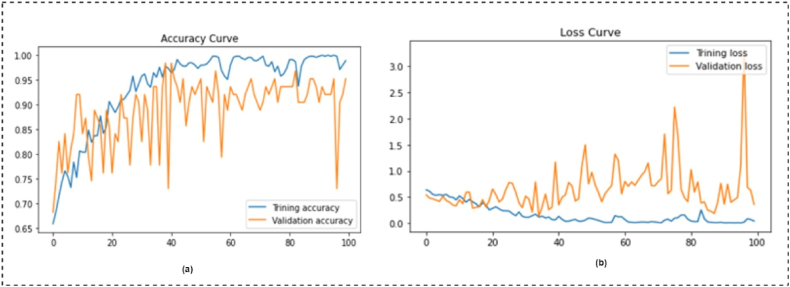
Loss curve and accuracy curve over 100 epochs. (a) Depicted the accuracy curve of the training and validation, (b) depicted the loss curve of the training and validation.

Fig. 17 shows the best performing model's loss and accuracy curves. Fig. 17 (a) shows the training and validation accuracy during the model learning. There are no bumps in the training curve from the first to the final epoch. Since the training and validation curves are closely converging with only a small gap between them, there was no apprehension of overfitting during network training. The model's training and validation loss is shown in Fig. 17 (b).

### 4.6 Statistical analysis of the best model

False discovery rate (FDR), false positive rate (FPR), false negative rate (FNR), kappa coefficient (KC), and Matthew's correlation coefficient (MCC) rates, and the efficacy in terms of error rates using RMSE and mean absolute error (MAE) for the best CNN model (RKONet-13), are enumerated in Table 12.

**Table 12:** Performance metrics of RKONet-13.

RKONet-13	FPR (%)	2.19
	FNR (%)	0.00
	FDR (%)	4.65
	KC (%)	96.5
	MCC (%)	96.44
	MAE (%)	2.01
	RMSE (%)	5.98

The false positive rate of 2.19 %, a false negative rate of 0, a false discovery rate of 4.65 %, a Kappa coefficient of 96.5 %, a Matthew correlation coefficient of 96.44 %, a mean absolute error of 2.01 %, and a root mean squared error of 5.98 %.

### 4.7 Comparison with transfer learning models

Five transfer learning models, VGG16 [84], VGG19 [1], ResNet50 [112], InceptionV3 [113,112], and AlexNet [113], are employed in this section to the same datasets.

### 4.8 Compare accuracy of transfer learning model between the raw dataset, preprocessed dataset, and the dataset after segmentation

A comparison of the proposed model's performance with the five other CNN-based transfer learning models is shown in Table 13. All the transfer learning models, and the RKONet-13 model were evaluated on the same datasets. The hyperparameters of the model were the same as described above. For all models, the image size is kept at 224 pixels, the Adam optimizer is used, and all models run for 100 epochs. A learning rate of 0.001 is used for all models. As compared to all other models, the developed RKONet-13 model performed the best in terms of accuracy.

**Table 13:** Comparison between the transfer learning models and proposed model.

Classifier Name	Raw Dataset		Pre-processed Dataset		Segmented Dataset	
	Test Accuracy	Test Loss	Test Accuracy	Test Loss	Test Accuracy	Test Loss
VGG16	74.83 %	0.181	84.81 %	0.181	94.13 %	0.181
VGG19	74.66 %	0.163	84.83 %	0.163	93.13 %	0.163
ResNet50	84.96 %	0.199	84.19 %	0.199	94.12 %	0.199
InceptionV3	74.01 %	0.191	85.36 %	0.191	92.93 %	0.191
AlexNet	74.17 %	0.191	83.17 %	0.191	92.13 %	0.191
Proposed Model	79.03 %	0.153	88.96 %	0.146	98.41 %	0.304

The results above demonstrate the importance of image preprocessing and segmentation. The accuracy of the models with the raw dataset was low, as there the raw image contained text, noise and artifacts. After removing text, noise, and artifacts from the raw images and the accuracy increased for all models. ROIs were then extracted by segmentation resulting in a further increase in accuracy for all models.

The raw dataset, the preprocessed dataset before segmentation, and the dataset after segmentation are loaded and run one by one to find the training, validation, and test accuracy as well as loss values. An overview of the performance analysis of the proposed model and the transfer learning model for the raw dataset, the pre-processed dataset before segmentation and the dataset after segmentation, is shown in Table 13.

### 4.9 Proposed model's performance analysis on breast mammogram dataset

As previously stated, this study emphasized an additional breast cancer mammogram dataset to verify the flexibility and efficiency of the proposed RKONet-13 model. Table 14 demonstration the performance of the model with mammogram dataset having four different classes.

**Table 14:** Performance of proposed model on Mammogram dataset.

Performance of the proposed Model with Mammogram Dataset	Accuracy	96.21 %
	Precision	96.00 %
	Recall	96.25 %
	F1 score	96.00 %

Table 14 shows that the proposed model provides remarkable performance on the additional mammogram dataset. In addition, the images of the dataset come from an entirely different modality and the dataset contains four classes. The proposed model demonstrates its capacity to accurately diagnose breast cancers, achieve 96.21 % accuracy with a high precision score of 96.00 %. The model achieves 96.25 % recall rate, and its 90.00 % F1 score denotes a harmonic balance between accuracy and recall. The resilience of the model and its potential for the precise categorization of breast tumor are highlighted by the combined effects of these findings.

### 4.10 K-fold cross validation

**Fig. 18:**
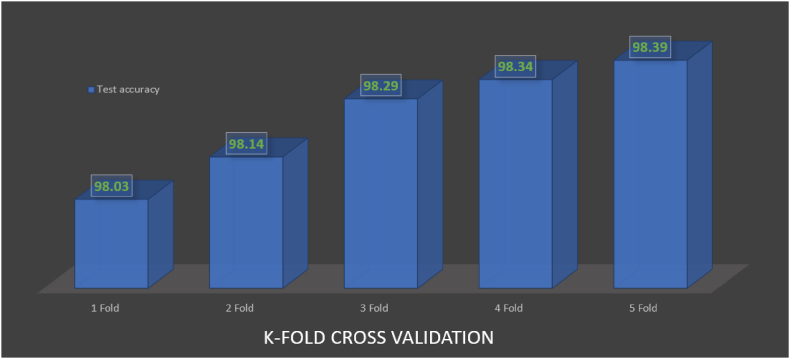
K-fold cross validation.

This study includes a five-fold cross-validation experiment to assess the robustness of the proposed model. A validation test known as K-Fold cross-validation is employed using the training and test datasets [114,115]. There will be k iterations of training and validation, with a different data fold for each. Using this technique, it is possible to see how variable bias and randomness affect accuracy, where the tendency is shown as a discrepancy between actual and anticipated accuracy. Fig. 18 shows the k-fold cross validation.

This study uses k-Fold cross-validation to assess the model's robustness, and stability with a 1-fold, 2-fold, 3-fold, 4-fold and 5-fold values. This resulted in a testing accuracy of 98.03 %, 98.14 %, 98.29 %, 98.34 %, and 98.39 %, respectively. The highest classification accuracy for the proposed model was 98.41 %.

### 4.11 Comparison with existing work

This section compares the models of ten research papers on breast cancer classification with the proposed model. The comparison is based on various factors, such as the dataset, the classifier employed, and the accuracy achieved. The findings of these studies are presented in Table 15.

**Table 15:** Comparison of the proposed model with existing work.

Paper	Dataset	Classifier	Accuracy
Irfan et al. [31]	BUI	Bi–CNN, transfer learning	98.9 %
Jianrui et al. [44]	Department of Ultrasound, Second Affiliated Hospital of Harbin Medical University	Self-organizing map, Machine Learning Classifier	91.07 %
Zhemin et al. [38]	1) BUSI 2) OMI 3) Dataset B 4) Hospital	adaptive spatial feature fusion	95.48 %
tariq et al. [62]	1) OASBUD 2) BHE	Ensemble Classifier, RUSBoost	99.98 % 97.86 %
Mishra et al. [63]	BUSI	ML Radiomics based Classifier	97.4 %
Kiran et al. [64]	BUSI	Transfer learning with feature fusion, RDE, RGW, Machine learning Classifier	99.1 %
Byra et al. [65]	BUSI	Hybrid Classifier (Transfer learning + scaling layers)	91.5 %
Moon et al. [66]	1) Private Dataset 2) BUSI	Ensemble CNN (VGGNet, ResNet, DenseNet)	91.10 % 94.62 %
Yang et al. [67]	Collect from 32 Hospital	Deep Learning	86.40 %
Xiangmin et al. [68]	Biomodal breast Ultrasound	DDSTN, MMD, LUPI	86.79 %
**Proposed Model**	**BUI**	**Shallow robust CNN**	**98.41 %**

In Table 15, a comparison is made between the proposed model for breast cancer classification and previous studies. The table shows the various studies, their datasets, the classifiers used, and the accuracy obtained. The analyses include Zhemin et al. used an adaptive spatial feature fusion classifier and achieved 95.48 % accuracy, Mishra et al. used an ML Radiomics-based classifier and reached 97.4 % accuracy, Byra et al. had used a Hybrid Classifier and achieved 91.5 % accuracy, Kiran et al. used a Machine learning Classifier and achieved 99.1 % accuracy, Moon et al. used an Ensemble CNN classifier and gained 91.10 % and 94.62 % accuracy, Tariq et al. used an Ensemble Classifier and achieved 99.98 % and 97.86 % accuracy, Jianrui et al. used a Machine Learning Classifier and performed 91.07 % accuracy, Yang et al. used a Deep Learning classifier and achieved 86.40 % accuracy, Irfan et al. used a Bi–CNN classifier and reached 98.9 % accuracy, and Xiangmin et al. used a DDSTN classifier and performed 86.79 % accuracy. The proposed model used a shallow robust CNN classifier and achieved 98.41 % accuracy.

## 5 Discussion and conclusion

This section presents the overall discussion of this study, the comprehensive findings obtained through this study, emphasizing the most significant outcomes of this study. Furthermore, the limitations of this study research and recommendations of future research are discussed.

### 5.1 Findings

The second-deadliest cancer of women is breast cancer [1]. The fatality rates can be considerably reduced by early diagnosis and treatment [11]. Breast cancer can be challenging to detect in the early stages. Breast cancer screening and diagnosis take a lot of time, and the presence of noise, artifacts, and other concerns like supply of medical equipment makes it difficult for a radiologist to categorize the medical pictures. In the COVID-19 pandemic situation, the medical supply chain had stopped, and it has led to several health consequences, such as depression, anxiety, and mental stress [116,117].In addition, there is a global shortage of medical equipment for covid 19, radiologists and medical professionals who can interpret the screening data, particularly in rural areas and developing nations. Computer-aided detection (CAD) systems can reduce radiologists' workloads by supporting them in the examination of breast ultrasound images. Computer-aided detection (CAD) systems reduce radiologists' workloads by supporting them in the examination of breast ultrasound images. As a result of recent innovations, artificial intelligence (AI) technologies have been developed for the automated diagnosis of breast tumors based on ultrasound images. For example, deep learning may benefit breast cancer picture analysis. This study aims to classify breast ultrasound images into benign and malignant by employing robust CNN model after segmenting the ROI (Reason of the Interest) employing a robust and optimized UNet architecture. Extracting the ROI is tough from ultrasound images due to lots of noise and artifacts. This study begins with image preprocessing like eliminating textural irregularities, unwanted noise, and artifacts from raw ultrasound images by meticulously applied to data preprocessing algorithms. The preprocess images undergo various statistical analysis to verify the image quality is not reduced. PSNR, SSIM, RMSE, and SSIM assess the degree of deterioration and resemblance to the source images to assess the success of the processing approaches. A novel architecture hybrid attention UNet (RKO-UNet) is introduced to solve the image segmentation. This model is built based on the base UNet architecture, which has proven to be effective at segmenting medical images. The RKO-UNet makes use of intricate spatial correlations in pictures while simultaneously boosting feature extraction across channels by including both spatial self-attention and channel self-attention techniques. A CNN model (RKONet-13) has been developed from based CNN model which build from a scratch. Both models have been refined through architectural and hyperparameter ablation study. The RKONet-13 provides optimal classifier performance for breast tumor detection on benign and malignant. This classification model provides remarkable accuracy and proficiency in detecting breast cancer also from diffract modalities like mammograms. To ensure the model's reliability this study conducted a five-fold validation, which proven the model's consistent performance. In one-word RKONet-13 proves to be highly effective and holds great promise in the realm of breast tumor detection.

### 5.2 Case study

The practical relevance of this study in real-life engineering can be seen in medical diagnostic settings where a breast tumor diagnosis tool for ultrasound images can be built incorporating the model and integrated into the hospital workflows. Companies actively embrace Corporate Social Responsibility (CSR) by maintaining business networks and entrepreneurial collaborations that promote knowledge-sharing and innovation. Companies that work with medical technologies can develop more advanced software solutions or may develop an advanced ultrasound machine that will capture internal images and give a decision at the same time for automated breast tumor analysis. The automated diagnostic systems may assist radiologists in accurately identifying and categorizing breast tumors from ultrasound images that will be potentially time saver, revolutionize breast tumor analysis and provide real-time automated diagnostic insights. This commitment to progress not only accelerates medical research but also underscores dedication to enhancing diagnostic precision with better performance.

### 5.3 Policy recommendation

Policy makers should prioritize AI-based techniques like RKONet-13 in order to detect and diagnose breast cancer earlier and more accurately. Research collaborations, funding initiatives, and training programs are important to ensure widespread adoption of AI-assisted tools for accurate and precise breast cancer classification, particularly in resource-constrained areas and during healthcare disruptions like COVID-19. It can help remote diagnosis and screening also reduce burden of hospital, radiologist also.

This study addresses the political factors when the RKO-UNet and RKONet-13 has been used in a wide range. These included navigating various regulatory frameworks, protecting patients’ privacy while the data has been used or shared, reducing the healthcare disparities through equitable access, fostering international collaboration. On the other hand, for transparent healthcare resource allocation, intellectual property issues addressing cross border technology transfer as well as overcoming public skepticism through education should be in concern.

### 5.4 Research limitation

The proposed segmentation (RKO-UNet) and CNN (RKONet-13) models performed significantly better than conventional models. However, the proposed model has some limitations that may be explored in the future like the small size of the dataset. On the other hand, it's important to note that this study did not use real-time medical images obtained directly from a hospital environment, but real time medical datasets could be used to dealing with some difficulties of challenges.

### 5.5 Recommendation for future research

The limitations of these studies are shown in the previous section. The main goal of the future scope will be to reduce or eliminate all the limitations that were in this study. Working with the different modalities of breast images like mammogram, ultrasound, and histopathological images. Ensuring a large number of real time medical images obtained directly from one or more hospitals is another future goal of this study. In the future this research must address the political issues when the RKONet-13 is widely used in the medical fields.

## 6 Conclusion

This study presents a novel segmentation and classification scheme for breast tumor ultrasound images. A segmentation model named RKO-UNet is introduced that is optimized through an ablation study. The segmentation is employed on pre-processed images, done for relevant and better input quality for the segmentation model. A CNN model named RKONet-13 is developed for the superior classification, integrating ablation study and attention unit. The result of the proposed RKONet-13 model is compared with five transfer learning models, and k-fold cross-validation is used to assess its performance stability. Several performance metrics show the effectiveness of the proposed technique. A mammogram dataset is also employed to classify breast cancer with the proposed model and the model gives a noteworthy performance. This study relies on image pre-processing, hybrid attention UNet, robust CNN, fine-tuning, and ablation study to achieve the highest accuracy in segmentation and classification and shows a promising performance in the diagnosis of breast cancer from ultrasound imaged. Moreover, RKO-UNet and RKONet-13 models advance the segmentation and classification of breast tumor from ultrasound images, introducing a promising path for advanced medical image analysis.

## Data availability statement

Dataset used in this study is publicly available form https://www.kaggle.com/datasets/aryashah2k/breast-ultrasound-images-dataset.

## Funding

The research does not have any external funding.

## CRediT authorship contribution statement

**Shahed Hossain:** Investigation, Methodology. **Sami Azam:** Conceptualization, Investigation, Methodology. **Sidratul Montaha:** Data curation, Investigation, Resources. **Asif Karim:** Data curation, Methodology, Project administration, Resources. **Sadia Sultana Chowa:** Data curation, Investigation, Resources, Validation. **Chaity Mondol:** Writing – original draft, Writing – review & editing. **Md Zahid Hasan:** Validation, Writing – original draft, Writing – review & editing. **Mirjam Jonkman:** Investigation, Methodology, Validation.

## Declaration of competing interest

The authors declare that they have no known competing financial interests or personal relationships that could have appeared to influence the work reported in this paper.
